# Highly Oxygenated Limonoids and Lignans from *Phyllanthus flexuosus*

**DOI:** 10.1007/s13659-014-0026-2

**Published:** 2014-07-22

**Authors:** Jian-Qiang Zhao, Yan-Ming Wang, Hong-Tao Zhu, Dong Wang, Sheng-Hong Li, Rong-Rong Cheng, Chong-Ren Yang, Yi-Fei Wang, Min Xu, Ying-Jun Zhang

**Affiliations:** 1 State Key Laboratory of Phytochemistry and Plant Resources in West China, Kunming Institute of Botany, Chinese Academy of Sciences, Kunming, 650201 People’s Republic of China; 2 University of Chinese Academy of Sciences, Beijing, 100049 People’s Republic of China; 3 Guangzhou Jinan Biomedicine Research and Development Center, Guangzhou, 510632 People’s Republic of China

**Keywords:** *Phyllanthus flexuosus*, Euphorbiaceae, Limonoids, Lignan glycosides, Antifeedant, Antiviral, Cytotoxicity

## Abstract

Two new highly oxygenated limonoids, flexuosoids A (**1**) and B (**2**), and three new arylnaphthalene lignan glycosides, phyllanthusmins D–F (**3**–**5**), were isolated from the roots of *Phyllanthus flexuosus*, in addition to three known lignans, phyllanthusmin C, arabelline, and (+)-diasyringaresinol. Their structures were elucidated on the basis of detailed spectroscopic analysis and chemical methods. Compounds **1** and **2**, two new decaoxygenated limonoids with a C-19/29 lactol bridge and heptaoxygenated substituents at C-1, C-2, C-3, C-7, C-11, C-17, and C-30, represent the second example of limonoids in the Euphorbiaceae family. Most of the isolates were tested for their antifeedant, anti-herpes simplex virus 1, and cytotoxic activities. The new limonoids **1** and **2** showed promising antifeedant activity against the beet army worm (*Spodoptera exigua*) with EC_50_ values of 25.1 and 17.3 μg/cm^2^, respectively. In addition, both of them displayed moderate cytotoxicity against the ECA109 human esophagus cancer cell line, along with the known lignan glycoside, phyllanthusmin C, with the IC_50_ values of 11.5 (**1**), 8.5 (**2**), and 7.8 (phyllanthusmin C) μM, respectively.

Two new highly oxygenated limonoids, flexuosoids A (**1**) and B (**2**), and three new arylnaphthalene lignan glycosides, phyllanthusmins D–F (**3**–**5**), were isolated from the roots of *Phyllanthus flexuosus*, along with three known lignans. Flexuosoids A (**1**) and B (**2**) showed promising antifeedant activity against the beet army worm (*Spodoptera exigua*) with EC_50_ values of 25.1 and 17.3 μg/cm^2^, respectively. In addition, both of them and the known lignan glycoside, phyllanthusmin C, displayed moderate cytotoxicity against the ECA109 human esophagus cancer cell line, with the IC_50_ values of 11.5, 8.5, and 7.8 μM, respectively.

**Electronic supplementary material:**

The online version of this article (doi:10.1007/s13659-014-0026-2) contains supplementary material, which is available to authorized users.

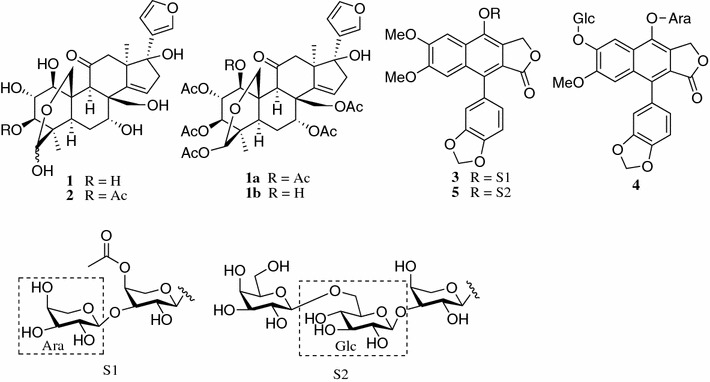

## Introduction

The genus *Phyllanthus* (Euphorbiaceae), comprising about 600 species, is widespread throughout the tropical and subtropical countries of the world, with about 30 species growing in the South of China. Several species have been used as traditional medicines [1], and flavonoids, alkaloids, sesquiterpenoids, triterpenoids, lignans, and tannins have been reported from the genus [2–6]. Among these, some sesquiterpenoids showed a wide range of biological properties including cytotoxic and antiviral effects [5–7].

*Phyllanthus flexuosus* (Sieb. et Zucc.) Muell. Arg (Euphorbiaceac), a shrub up to 3 m height, mainly grows in the southern parts of the Yangtze River, People’s Republic of China. The whole plants have been utilized medicinally for treating infectious diseases by the Dai people in Yunnan Province, China [8]. Chemical studies on the leaves and stem barks of *P.flexuosus* have revealed the occurrence of triterpenoids, tannins and coumarins [9–12]. Previously we obtained three phenylacetylene-bearing tricyclic diterpenes from this species, among which phyllanflexoids A was the first example of phenylacetylene-bearing 18-nor-diterpenoid glycoside [13]. As a part of our continuing research for new bioactive secondary metabolites from the genus *Phyllanthus* [6, 14–17], five new compounds, including two highly oxygenated limonoids, flexuosoids A (**1**) and B (**2**), and three arylnaphthalene lignan glycosides, phyllanthusmins D–F (**3**–**5**), were isolated from the roots of *P. flexuosus*, together with three known lignans. Their structures were elucidated by extensive spectroscopic analysis and chemical method. Most of the isolated compounds were evaluated for their antifeedant, anti HSV-1, and cytotoxic activities, and the results obtained are discussed herein.

## Results and Discussion

**Fig. 1:**
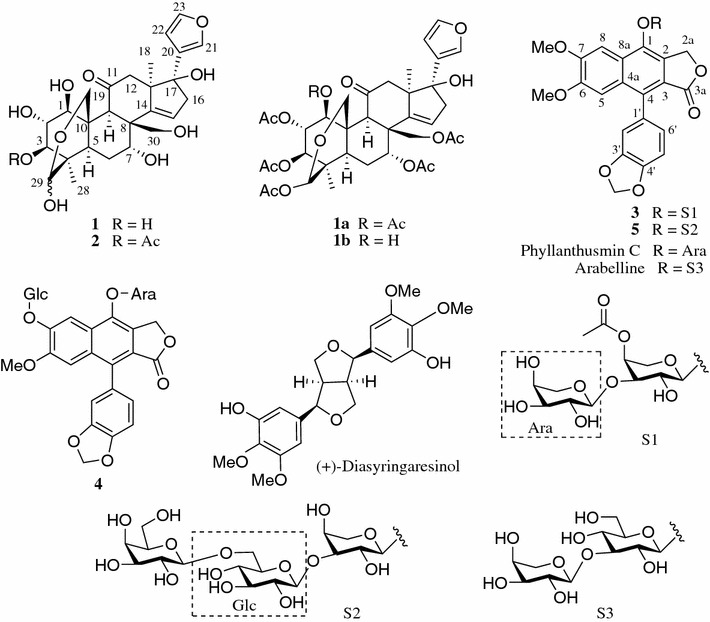
Structures of compounds from the roots of *P. flexuosus*

The MeOH extract of the air-dried roots of *P. flexuosus* was applied to repeated column chromatography over macroporous resin D101, Sephadex LH-20 and silica gel, followed by semi-preparative HPLC, to afford five new compounds (**1–5**), in addition to three known lignans. The known compounds were identified as phyllanthusmin C [18], arabelline [19], and (+)-diasyringaresinol [20] by comparison of their spectroscopic data with literature values (Fig. 1).

**Fig. 2:**
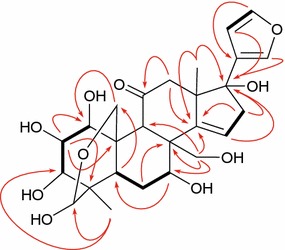
Key ^1^H-^1^H COSY (**–**) and HMBC () correlations of **1**

Compound **1** was obtained as a white amorphous powder. The negative HRESIMS (*m/z* 505.2072 [M − H]^−^, calcd for C_26_H_33_O_10_, 505.2073) and the ^13^C NMR (DEPT) spectra of **1** indicated a molecular formula of C_26_H_34_O_10_, requiring for 10 degrees of unsaturation. The IR spectrum of **1** indicated the presence of hydroxy (3424 cm^−1^) and carbonyl (1691 cm^−1^) groups. The ^13^C NMR (DEPT) data for **1** (Table 1) revealed 26 carbon signals, including characteristic signals due to a hemiacetal ring unit (*δ*_C_ 97.36 and 64.81) [21], a *β*-substituted furan ring (*δ*_C_ 128.55, 141.25, 110.63 and 144.33) [22], a trisubstituted double bond (*δ*_C_ 153.48 and 121.76) and a ketone (*δ*_C_ 214.73). The hemiacetal ring [H_2_-19: *δ*_H_ 4.20 and 4.52 (each 1H, *J* = 12.9 Hz); H-29: *δ*_H_ 4.83] [21] and the *β*-substituted furan ring protons (*δ*_H_ 6.42, 7.52, 7.47) [22] were also distinguished from the ^1^H NMR spectrum (Table 2), in addition to two methyl singlets at *δ*_H_ 0.97 and 1.03. The aforementioned NMR data for **1** were closely related to those of 3*α*-deacetylamoorastatin [23]. However, a major difference between these two compounds was observed as the oxidation of a methyl and a methylene in 3*α*-deacetyl-amoorastatin core to an oxymethylene (*δ*_C_ 71.92, *δ*_H_ 3.60, 3.89 (each 1H, d, *J* = 10.7 Hz) and an oxymethine [*δ*_C_ 68.60, *δ*_H_ 4.54 (dd, *J* = 4.4, 3.4 Hz] in **1**, respectively. Moreover, the 14,15-epoxide and the C-17 methane in 3*α*-deacetyl-amoorastatin were replaced with a Δ^14(15)^ double bond (*δ*_C_ 153.48 and 121.76) and an oxygen-bearing quaternary carbon (*δ*_C_ 84.21, C-17) in **1**, respectively. The above data were further assigned unambiguously by HSQC, ^1^H-^1^H COSY and HMBC analysis (Fig. 2 and Electronic supplementary material). The ^1^H-^1^H COSY spectrum verified the presence of -CH(O)(1)–CH(O)(2)–CH(O)(3)–, –CH(5)–CH_2_(6)–CH(O)(7)–, and =CH(15)–CH_2_(16) fragments in **1** (bold lines in Fig. 2). The HMBC correlations (Fig. 2) from the oxymethylene at *δ*_H_ 3.60 and 3.89 (H_2_-30) to *δ*_C_ 69.54 (C-7)/*δ*_C_ 52.89 (C-8)/*δ*_C_ 46.06 (C-9)/*δ*_C_ 153.48 (C-14), and from the oxymethine at *δ*_H_ 4.54 (H-2) to *δ*_C_ 75.84 (C-1)/*δ*_C_ 43.96 (C-4)/*δ*_C_ 43.41 (C-10) indicated that C-30 and C-2 of **1** were oxygenated, respectively. The Δ^14(15)^ double bond was confirmed by the HMBC correlations of H_2_-12, H_2_-16 and Me-18 to the olefinic carbon at *δ*_C_ 153.48 (C-14). Moreover, HMBC correlations from H-15, H_2_-16, Me-18, H-21 and H-22 to the oxygen-bearing quaternary carbon (*δ*_C_ 84.21, C-17) revealed the hydroxylated C-17 of D ring, on which the furan ring was located in **1**. Other HMBC correlations confirmed the planar structure of **1** as shown.

**Table 1:** ^13^C NMR spectroscopic data of **1** and **2** (methanol-*d*_4_, *δ* in ppm)

Position	1^a^		2^a^	
	29-*exo*	29-*endo*	29-*exo*	29-*endo*
1	75.84, CH	75.76, CH	74.36, CH	74.36, CH
2	68.60, CH	68.81, CH	67.88, CH	67.99, CH
3	75.78, CH	79.27, CH	76.69, CH	79.83, CH
4	42.96, C	43.17, C	42.65, C	42.58, C
5	27.69, CH	25.06, CH	29.15, CH	27.76, CH
6	25.62, CH_2_	27.77, CH_2_	25.70, CH_2_	26.67, CH_2_
7	69.54, CH	69.79, CH	69.61, CH	69.88, CH
8	52.89, C	52.95, C	52.96, C	53.01, C
9	46.06, CH	46.36, CH	46.06, CH	46.39, CH
10	43.41, C	43.51, C	43.41, C	43.41, C
11	214.73, C	214.89, C	214.71, C	214.89, C
12	44.31, CH_2_	44.31, CH_2_	44.37, CH_2_,	44.52, CH_2_
13	53.03, C	52.96, C	53.15, C	52.90, C
14	153.48, C	153.55, C	153.55, C	153.60, C
15	121.76, CH	121.69, CH	121.89, CH	121.80, CH
16	44.45, CH_2_	44.39, CH_2_	44.59, CH_2_	44.45, CH_2_
17	84.21, C	84.21, C	84.33, C	84.33, C
18	26.12, CH_3_	26.05, CH_3_	26.21, CH_3_	26.15, CH_3_
19	64.81, CH_2_	59.49, CH_2_	65.17, CH_2_	59.32, CH_2_
20	128.55, C	128.55, C	128.79, C	128.79, C
21	141.25, CH	141.25, CH	141.34, CH	141.34, CH
22	110.63, CH	110.63, CH	110.73, CH	110.73, CH
23	144.33, CH	144.33, CH	144.42, CH	144.42, CH
28	20.45, CH_3_	19.73, CH_3_	19.75, CH_3_	19.32, CH_3_
29	97.36, CH	97.09, CH	96.88, CH	96.80, CH
30	71.92, CH_2_	72.01, CH_2_	72.04, CH_2_	72.08, CH_2_
COMe			173.31, C	173.25, C
COMe			21.23, CH_3_	21.18, CH_3_

^a^Data were measured at 125 MHz

**Table 2:** ^1^H NMR spectroscopic data of **1** and **2** (methanol-*d*_4_, *δ* in ppm)

Position	1^a^		2^a^	
	29-*exo*	29-*endo*	29-*exo*	29-*endo*
1	4.35, d (4.4)	4.35, d (4.4)	4.37, d (4.6)	4.37, d (4.6)
2	4.54, dd (4.4, 3.4)	4.50^b^	4.59^b^	4.54^b^
3	3.93, d (3.4)	3.42, d (3.2)	5.50, d (4.8)	4.97, d (4.8)
5	2.63, dd (13.9, 3.0)	2.48^b^	2.64, dd (10.9, 2.9)	2.71^b^
6	1.78, dd (13.7, 3.5) 1.90, br. d (14.5)	1.81, br. d 2.48^b^	1.76, dd (10.9, 3.5) 1.88, br. d (14.1)	1.78^b^ 2.45^b^
7	4.05, br. d	4.02, br. d	4.04, br. d	4.00, br. d
9	4.01, s	4.03, s	4.01, s	4.03, s
12	1.99, d (19.2) 3.25, d (17.9)	1.99, d (19.2) 3.25, d (17.9)	3.25, d (19.3) 1.89, d (19.1)	3.25, d (19.3) 1.89, d (19.1)
15	5.73, br. d	5.73, br. d	5.73, br. d	5.73, br. d
16	2.52, dd (16.3, 3.3) 3.25, d (17.9)	2.52, dd (16.3, 3.3) 3.25, d (17.9)	2.51, dd, (16.3, 3.5) 3.24, d (16.2)	2.51, dd, (16.3, 3.5) 3.24, d (16.2)
18	1.03, s	1.03, s	1.04, s	1.02, s
19	4.20, d (12.9) 4.52, d (12.9)	4.52^b^ 4.56^b^	4.18, d (10.2) 4.56^b^	4.38^b^ 4.38^b^
21	7.52, s	7.52, s	7.53, s	7.53, s
22	6.42, s	6.42, s	6.43, s	6.43, s
23	7.47, s	7.47, s	7.47, s	7.47, s
28	0.97, s	1.00, s	0.82, s	0.83, s
29	4.83, s	4.62, s	4.80, s	4.72, s
30	3.60, d (10.7) 3.89, d (10.7)	3.61, d (9.8) 3.93, d (9.8)	3.60, d (11.1) 3.87, d (11.1)	3.63^b^ 3.92, d (11.1)
COMe			2.09, s	2.08, s

^a^Data were measured at 500 MHz

^b^Overlapping ^1^H NMR signals are reported without designated multiplicity

**Fig. 3:**
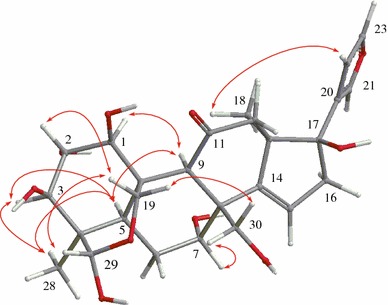
Key ROESY () correlations of **1**

The relative stereochemistry of **1** was fixed by the proton coupling constants observed in the NMR spectrum and ROESY experiment (Fig. 3). The ROESY correlations of H-5/H-9, H-1/H-9, H-5/Me-28, H-3/Me-28, H-3/H-5, and H-2/H-19 supported the relative configuration of the fused A/B rings as shown in Fig. 1, and the hemiacetal ring between C-19 and C-29 was on the same side to those of 1-OH and 3-OH. The small values of *J*_1,2_ (4.4 Hz) and *J*_2,3_ (3.4 Hz) indicated the equatorial H-1, H-2 and H-3, corresponding to *β*-configurations for 1-OH, and 3-OH, and *α*-configurations for 2-OH. In the ^1^H NMR spectrum of **1**, the broad singlet H-7 suggested an *α*-configuration of 7-OH, which was further confirmed by ROESY correlations of H-29/H-19, H-19/H-30 and H-30/H-7. The 17-OH was assigned as *β* by a ROESY correlation of H-22 with Me-18. In addition, it is noted that compound **1** was isolated as a mixture of two tautomers in a ratio of 4:1 as estimated by ^1^H NMR spectrum. The ^1^H and ^13^C NMR spectral features of the mixture (Tables 1, 2) showed two sets of H-atom and C-atom resonances (partially overlapped) [24]. This could be explained by the equilibrium due to the hemiacetal unit. Acetylation of **1** yielded the 1,2,3,7,29,30-hexa-acetylated (**1a**) and 2,3,7,29,30-penta-acetylated (**1b**) adducts of **1**, whose structures (Fig. 1) were characterized by detailed spectroscopic analysis (see Electronic supplementary material). The 29-*exo* configurations for both **1a** and **1b** were supported by their chemical shifts of H-3 (*δ*_H_ 5.41), since H-3 resonated at *δ*_H_ 4.9–5.1 for 29-*endo* and 5.3–5.6 for 29-*exo* [25]. Based on the above evidence, compound **1** was determined as shown in Fig. 1 and named as flexuosoid A.

Compound **2** was obtained as a white amorphous powder and possessed a molecular formula C_28_H_36_O_11_, as deduced from the HRESIMS (*m/z* 547.2182 [M − H]^−^). The ^1^H and ^13^C NMR data of **2** were similar to those of **1**, except for the appearance of an additional acetyl group [*δ*_H_ 2.08/2.09 (3H, s) and *δ*_C_ 173.31/173.25 (CO), 21.23/21.18 (CH_3_)] and the down-field shift of C-3 and H-3 to *δ*_C_ 76.69 and *δ*_H_ 5.50, respectively, suggesting that the hydroxy group at C-3 in **1** was acetylated in **2**. This was confirmed by HMBC correlation of *δ*_H_ 5.50 (H-3) with the acetyl carbonyl carbon at *δ*_C_ 173.31 (Fig. 1S, Electronic supplementary material). In the ROESY spectrum, correlations of H-5/Me-28, H-5/H-9, H-19/H-29 and H-22/Me-18 supported the relative configurations of rings system of **2** as shown in Fig. 1. Moreover, the broad singlet H-7 in the ^1^H NMR spectrum and the ROESY correlations of H-30/H-7 of **2** revealed that 7-OH had the *α*-configuration. The ROESY correlation of H-22 with Me-18 supported the assignment of the *β*-configuration to 17-OH, and the correlations of H-1/H-9 and H-3/H-28 relieved the *β*-configuration to 1-OH and 3-OH. Due to the severe overlapping of H_2_-19 and H-2 signals in **2**, the assignment of *α*-orientation for hydroxy group at C-2 was achieved by acetylation of **2** to give 1,2,3,7,29,30-hexa-acetyl flexuosoid A (**1a**) and 2,3,7,29,30-penta-acetyl flexuosoid A (**1b**). In the ^1^H NMR spectrum of **1a**, the small values of *J*_1,2_ (4.4 Hz) and *J*_2,3_ (4.4 Hz) revealed the equatorial H-1, H-2, and H-3 in **2**. Furthermore, the 29-*exo* configuration for **2** was supported by chemical shifts of H-3 of **1a** and **1b**, the acetylated products of **2**. Thus, **2** was assigned as shown in Fig. 1 and named as flexuosoid B.

**Fig. 4:**
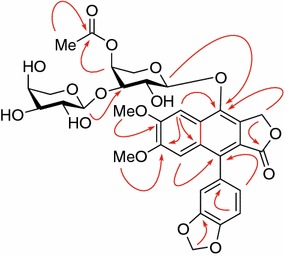
Key HMBC () correlations of **3**

Compound **3**, a white amorphous powder, possessed a molecular formula C_33_H_34_O_16_, as deduced by the HRESIMS at *m/z* 685.1763 [M − H]^−^ (calcd for C_33_H_33_O_16_, 685.1768). The IR spectrum showed the presence of hydroxy (3423 cm^−1^) and benzene rings (1622, 1507, and 1435 cm^−1^). The ^13^C NMR and DEPT spectra (Table 3) displayed the presence of 33 carbon resonances, due to two carboxyls (*δ*_C_ 172.09 and 172.5), 16 aromatic carbons (*δ*_C_ 102–153), one methylenedioxy (*δ*_C_ 102.6), one oxymethylene (*δ*_C_ 69.0), two methoxys (*δ*_C_ 56.7 and 56.0), two pentosyl moieties and an acetyl methyl (*δ*_C_ 21.2). In the ^1^H NMR spectrum, two singlet aromatic protons at *δ*_H_ 6.89 and 8.03 (each 1H, s) and a set of ABX coupled signals [*δ*_H_ 6.87 (1H, d, *J* = 8.0 Hz), 6.64 (1H, d, *J* = 1.5 Hz), 6.59 (1H, dd, *J* = 8.0, 1.5 Hz)] arising from an 1,3,4-trisubstituted benzene ring were observed, in addition to characteristic proton signals due to a methylenedioxy (*δ*_H_ 6.02 and 6.00, each 1H, s), two methoxys [*δ*_H_ 3.94, 3.63 (each 3H, s)], and two anomeric protons [*δ*_H_ 4.74 (d, *J* = 7.7 Hz) and 4.49 (d, *J* = 7.0 Hz)]. Acid hydrolysis of **3** afforded L-arabinose as the soly monosaccharide, which was confirmed by GC analysis of its corresponding trimethylsilylated l-cysteine adducts. The aforementioned data of **3** were closely related to those of phyllanthusmin C, a known arylnaphthalene lignan glycoside from *Phyllanthus oligospermus* [18]. The major difference was the presence of one more arabinosyl and an additional acetyl group in **3**, compared with phyllanthusmin C. All signals of **3** were assigned completely on the basis of HSQC, HMBC and ^1^H-^1^H COSY experiments. In the HMBC spectrum of **3** (Fig. 4), correlation of an anomeric proton at *δ*_H_ 4.74 with C-1 (*δ*_C_ 146.1) confirmed the linkage of an arabinosyl moiety at C-1 of lignan aglycone. Subsequently, HMBC correlations of the additional arabinosyl anomeric proton at *δ*_H_ 4.49 with the inner arabinosyl C-3′ (*δ*_C_ 81.8) and the inner arbinosyl H-4′′ (*δ*_H_ 5.16) with the acetyl carbonyl carbon (*δ*_C_ 172.5), indicated that the additional arabinosyl and acetyl units were linked to the inner arabinosyl C-3′ and C-4′ in **3**, respectively. This resulted in deshielded resonances of the inner arabinosyl C-3′ and C-4′ of **3** by 7.4 and 2.8 ppm, respectively, and shielded resonances of C-5′ by 2.2 ppm, compared with those of phyllanthusmin C. On the basis of these observations and other HMBC correlations (Fig. 4 and Electronic supplementary material), the structure of phyllanthusmin D (**3**) was established.

**Table 3:** ^1^H and ^13^C NMR spectroscopic data of **3** and **4** (methanol-*d*_4_, *δ* in ppm)

Position	3^a^		4^b^	
	*δ*_H_ (*J* in Hz) *δ*_C_		*δ*_H_ (*J* in Hz) *δ*_C_	
1		146.14/146.13^c^, C		146.5, C
2		119.85/119.76^c^, C		120.71/120.72^c^, C
3		132.19/132.10^c^, C		132.70/132.72^c^, C
4		137.47/137.40^c^, C		137.5, C
4a		131.69/131.66^c^, C		132.3, C
5	6.89^c^, s	106.75/106.70^c^, CH	7.13^c^,s	107.7, CH
6		151.51/151.47^c^, C		151.7, C
7		153.14/153.11^c^, C		150.6, C
8	8.03, s	102.63/102.59^c^, CH	8.25, s	106.5, C
8a		128.64/128.61^c^, C		128.5, C
1′		129.74/129.80^c^, C		130.2, C
2′	6.64, d (1.5)	111.65/111.71^c^, CH	6.81^c^, d (1.7)	111.86/111.89^c^, CH
3′		148.86/148.90^c^, C		149.2, C
4′		148.81/148.90^c^, C		149.2, C
5′	6.87^c^, d (8.0)	108.84/108.88^c^, CH	6.96^c^, d (7.8)	109.18/109.20^c^, CH
6′	6.59^c^, dd (8.0, 1.5)	124.71/124.74^c^, CH	6.77^c^, dd (7.8, 1.7)	124.9, CH
2a	5.48^c^, d (15.2) 5.39^c^, d (15.2)	69.0, CH_2_	5.47^c^, d (15.1) 5.62^c^, d (15.1)	69.4, CH_2_
3a		172.09/172.05^c^, C		172.3, C
6-OMe	3.63^c^, s	56.0, CH_3_		
7-OMe	3.94, s	56.7, CH_3_	3.76, s	56.3, CH_3_
O–CH_2_–O	6.02^c^, s 6.00^c^, s	102.6, CH_2_	6.06^c^, s 6.05^c^, s	102.8, CH_2_
1″	4.74, d (7.7)	106.9, CH	4.76^c^, d (7.4)	107.3, CH
2″	4.12, dd (8.6, 7.7)	72.2, CH	3.99, dd (9.3, 7.4)	72.9, CH
3″	3.83^d^	81.8, CH	3.65, dd (9.3, 3.5)	74.4, CH
4″	5.16, br. s	72.5, CH	3.86, br. s	70.0, CH
5″	3.98^d^ 3.52^d^	65.4, CH_2_	3.50, d (12.5) 3.93, dt (12.5, 1.9)	67.8, CH_2_
1‴	4.49, d (7.0)	106.5, CH	5.31, d (7.5)	101.3, CH
2‴	3.66, dd (8.8, 7.0)	72.6, CH	3.60, dd (9.2, 7.6)	74.9, CH
3‴	3.54^d^	74.1, CH	3.55, t (9.2)	78.2, CH
4‴	3.81, br. s	69.5, CH	3.41, t (9.2)	71.5, CH
5‴	3.86^d^ 3.52^d^	67.0, CH_2_	3.75^d^	78.0, CH
6‴			3.70, dd (11.9, 6.2) 3.97, dd (11.9, 2.0)	62.8, CH_2_
COMe		172.5, C		
COMe	2.15, s	21.2, CH_3_		

^a^Data were measured at 500 and 125 MHz for ^1^H and ^13^C, respectively

^b^Data were measured at 600 and 150 MHz for ^1^H and ^13^C, respectively

^c^Existing as very close pairs at room temperature (21 °C)

^d^Overlapping ^1^H NMR signals are reported without designated multiplicity

Compound **4** possessed a molecular formula of C_31_H_32_O_16_, on the basis of HRESIMS (*m/z* 661.1766 [M + H]^+^). Acid hydrolysis of **4** afforded L-arabinose and d-glucose as sugar residue, which was confirmed by GC analysis of its corresponding trimethylsilated l-cysteine adducts. The ^1^H and ^13^C NMR spectroscopic data (Table 3) of **4** showed close resemblance with those of phyllanthusmin C, except for the presence of only one methoxy group and an additional set of signals assignable to a *β*-glucosyl moiety [*δ*_H_ 5.31 (d, *J* = 7.5 Hz, *δ*_C_ 101.3, 74.9, 78.2, 71.5, 78.0 and 62.8] in **4**. In the HMBC experiment (Fig. 2S, Electronic supplementary material), correlations of H_2_-2a [*δ*_H_ 5.47, (d, *J* = 15.1); 5.62, (d, *J* = 15.1)] and *δ*_H_ 8.25 (H-8) with the quaternary carbon at *δ*_C_ 146.5 (C-1) allowed assignment of C-1 and H-8. The HMBC correlation from the arabinosyl anomeric proton (*δ*_H_ 4.76, Ara-1″) to C-1 (*δ*_C_ 146.5) indicated that the arabinosyl moiety was attached to C-1 of the lignan core. Moreover, HMBC correlation of the anomeric proton (*δ*_H_ 5.31, Glc-1‴) with C-7 (*δ*_C_ 150.6) supported linkage of the additional glucosyl moiety to C-7, which was further confirmed by the ROESY correlation between the anomeric proton (*δ*_H_ 5.31, Glc-1‴) with H-8 (*δ*_H_ 8.25). On the basis of these observations, the structure of **4** was established as phyllanthusmin E.

Compound **5** was isolated as a white amorphous powder. Its molecular formula was deduced to be C_38_H_44_O_21_ by HRESIMS (*m/z* 871.2077 [M − H]^−^). Acid hydrolysis of **5** afforded L-arabinose, d-glucose and d-galactose as sugar residue, which was confirmed by GC analysis of their corresponding trimethylsilylated l-cysteine adducts. The ^1^H and ^13^C NMR data (Table 4) of **5** were very similar to those of phyllanthusmin C, except for the appearance of two additional hexosyl units attributable to one glucosyl [*δ*_H_ 5.55 (d, *J* = 7.9 Hz), *δ*_C_ 105.5, 75.3, 79.0, 72.3, 78.9 and 68.6] and one galactosyl [*δ*_H_ 5.02 (d, *J* = 7.4 Hz), *δ*_C_ 106.2, 73.0, 75.5, 70.2, 76.9 and 63.1] units [26]. Linkage of the additional glucosyl unit to the arabinosyl C-3″ position was confirmed by the long range correlations between the glucosyl anomeric proton at *δ*_H_ 5.55 (H-1‴) and the arabinosyl C-3″ (*δ*_C_ 81.0). Moreover, HMBC correlation of the galactosyl H-1‴ (*δ*_H_ 5.02) with the glucosyl C-6‴ (*δ*_C_ 68.6) was also observed (Fig. 2S, Electronic supplementary material). Therefore, the structure of phyllanthusmin F (**5**) was elucidated as shown in Fig. 1.

**Table 4:** ^1^H and ^13^C NMR spectroscopic data of **5** (methanol-*d*_4_, *δ* in ppm)^a^

Position	*δ*_H_ (*J* in Hz)	*δ*_C_	Position	*δ*_H_ (*J* in Hz)	*δ*_C_
1		146.60/146.63^b^, C	O–CH_2_–O	5.93, s	102.3, CH_2_
2		120.46, C		6.03, s	
3		132.30/132.36^b^, C	Ara-1″	5.35, d (7.7)	107.4, CH
4		136.7, C	2″	4.26^c^	75.6, CH
4a		131.5, C	3″	4.59^c^	81.0, CH
5	7.36, s	107.4, CH	4″	4.01, br. d (9.5)	75.7, CH
6		151.6, C	5″	4.63^c^ 4.75, t (9.5)	68.9, CH_2_
7		153.2, C	Glc-1‴	5.55, d (7.9)	105.5, CH
8	8.85, s	103.3, CH	2‴	4.08^c^	75.5, CH
8a		128.9, C	3‴	4.39, t (8.3)	79.0, CH
1′		129.99/130.02^b^, C	4‴	4.22^c^	72.3, CH
2′	7.17/7.25, d (1.4)	112.09/112.20^b^, CH	5‴	4.22^c^	78.9, CH
3′		148.6, C	6‴	4.24^c^	68.6, CH_2_
4′		148.5, C		3.68, d (11.3)	
5′	7.16/7.14, d (7.8)	125.0, CH	Gal-1″″	5.02, d (7.4)	106.2, CH
6′	7.13/7.08, dd (7.8, 1.4)	109.0, CH	2″″	4.49, t (8.2)	73.0, CH
2a	5.70, dd (15.2, 3.7)	68.6, CH_2_	3″″	4.06^c^	75.1, CH
	6.11, dd (15.7, 10.9)		4″″	4.22^c^	70.2, CH
3a		170.7, C	5″″	4.28^c^	76.9, CH
6-OMe	3.66, s	55.9, CH_3_	6″″	4.56, d (16.3)	63.1, CH_2_
7-OMe	4.17, s	56.9, CH_3_		4.30^c^	

^a^Data were measured at 600 and 150 MHz for ^1^H and ^13^C, respectively

^b^Existing in pairs at room temperature (21 °C)

^c^Overlapping ^1^H NMR signals are reported without designated multiplicity

It was noted that the ^1^H and ^13^C NMR spectra of the new arylnaphthalene lignans (**3**–**5**) recorded at room temperature exhibited doubling of the signals, of which most aromatic signals existed as very close pairs, whose chemical shifts are different only at the second decimal places, due to the equilibrium between the two conformational isomers resulting from the slow rotation of sugar unit at room temperature around the glycosidic linkage [19]. The energy barrier about the glycosidic bond was sufficiently high to prevent fast exchange between the two rotamers at room temperature [27].

Most of the isolated compounds were tested for their antifeedant, anti HSV-1 and cytotoxic activities. The two new limonoids **1** and **2** displayed promising antifeedant activity against *Spodoptera exigua* with EC_50_ values of 25.1 and 17.3 μg/cm^2^, respectively, which were less active than that of the positive control (EC_50_ = 3.7 μg/cm^2^), a commericial neem oil containing 1 % azadirachtin, while slightly more active than aphanamixoid A (EC_50_ = 25.8 μg/cm^2^), a potential natural product with antifeedant activity [28]. Furthermore, both compounds **1** and **2**, together with the known lignan, phyllanthusmin C, showed moderate cytotoxicity against the ECA109 cell line, with the IC_50_ values of 11.5, 8.5, and 7.8 μM, respectively, compared to the positive control, 17-AAG (IC_50_ = 1.1 μM). However, all the tested compounds showed no inhibitory effects against HSV-1.

Limonoids, mainly found in the Meliaceae and Rutaceae families, are of rare occurrence in the Euphorbiaceae family [29]. The new deca-oxygenated flexuosoids A (**1**) and B (**2**) represent the second example of limonoids in the Euphorbiaceae family [30]. It is noteworthy that the new limonoids **1** and **2** bear a C-19/29 lactol bridge and oxygen substituents at C-1, C-2, C-3, C-7, C-11, C-17, and C-30 positions, of which the oxygen substituents at C-17 and C-30 are of rare occurrence among the reported limonoids [29]. Furthermore, compounds **1** and **2** showed not only promising antifeedant activity but also moderate cytotoxicity against the ECA109 human esophagus cancer cell line.

### Experimental Section

#### General Experimental Procedures

Optical rotations were performed on a P-1020 polarimeter (JASCO, Tokyo, Japan). IR spectra were measured on a Bruker Tensor 27 spectrometer with KBr pellets. 1D and 2D NMR spectra were run on Bruker DRX-500 and AV-600 instruments operating at 500 and 600 MHz for ^1^H, and 125 and 150 MHz for ^13^C, respectively. Coupling constants are expressed in Hertz and chemical shifts are given on a ppm scale with tetramethylsilane as internal standard. The MS data were recorded on a VG Auto Spec-3000 spectrometer (VG, Manchester, U.K.) with glycerol as the matrix. HRESIMS were recorded on an API Qstar Pulsa LC/TOF spectrometer. GC analysis was run on a Shimadzu GC-14C gas chromatograph. Column chromatography (CC) was performed with macroporous resin D101 (Mitsubishi Chemical Industry, Ltd., Tokyo, Japan), silica gel (200–300 mesh, Qingdao Haiyang Chemical Co., Ltd., Qingdao, People’s Republic of China), Sephadex LH-20 (25–100 μm, Pharmacia Fine Chemical Co. Ltd. Japan). Thin-layer chromatography (TLC) was carried out on silica gel H-precoated plates (Qingdao Haiyang Chemical Co., Ltd., Qingdao, People’s Republic of China) with CHCl_3_/MeOH/H_2_O (8.5:1.5:0.1, 8:2:0.2 or 7:3:0.5, v/v). Spots were detected by spraying with 10 % H_2_SO_4_ in EtOH followed by heating. Semi-preparative HPLC separation was performed on an Agilent 1260 liquid chromatography with a 5 μm Waters Sunfire-C18 column (10 × 250 mm, Waters, Sunfire TM, USA). GC analysis was run on Agilent Technologies HP5890 gas chromatography equipped with an H_2_ flame ionization detector. The column was 30QC2/AC-5 quartz capillary column (30 m × 0.32 mm) with the following conditions: column temperature: 180–280 °C; programmed increase, 3 °C/min; carrier gas: N_2_ (1 mL/min); injection and detector temperature: 250 °C; injection volume: 4 μL, split ratio: 1/50.

#### Cell Lines and Biochemicals

African green monkey kidney cells (Vero, ATCC CCL81), provided by Wuhan Institute of Virology, Chinese Academy of Sciences, were propagated in DMEM supplemented with 10 % heat-inactivated FBS. The constituents of the maintenance medium were the same as those of the growth medium except that only 2 % FBS was added. The cells were cultured at 37 °C in a humid atmosphere with 5 % CO_2_. HSV-1 strain F (ATCC VR-733), obtained from Hong Kong University, was propagated in Vero cells and stored at −80 °C until use. The human esophagus cancer cell ECA109 was cultured in RPMI 1640 containing 10 % heat inactivated FBS and 100 U/mL penicillin/streptomycin in a humidified incubator in a 5 % CO_2_ atmosphere at 37 °C.

### Plant Material

The roots of *P. flexuosus* were collected from Yunnan Province, People’s Republic of China, in June 2010. Voucher specimens (HITBC_015077) were deposited at the State Key Laboratory of Phytochemistry and Plant Resources in West China, Kunming Institute of Botany, Chinese Academy of Sciences, and were identified by Mr. Shi-Shun Zhou from Xishuangbanna Tropical Botanical Garden, Chinese Academy of Sciences.

### Extraction and Isolation

The air-dried and powdered roots of *P. flexuosus* (2.0 kg) were extracted with MeOH (3 times, 3 h each time) under reflux at 60 °C. The methanol extracts (320 g) were subjected to macroporous resin D101 column chromatography (CC) and eluted with the following gradient: MeOH/H_2_O (3:7, 6:4, 9:1) and finally MeOH, to give 4 fractions (Fr. 1–4). Fr. 2 (45.0 g) and Fr. 3 (16.6 g) were subjected separately to repeated CC on silica gel (CHCl_3_/MeOH/H_2_O, 9:1:0.1–7:3:0.5) and Sephadex LH-20 (MeOH/H_2_O, 1:9–8:2) to yield **3** (13 mg), phyllanthusmins C (20 mg), arabelline (12 mg), (+)-diasyringaresinol (8 mg) and subfraction F2A (32 mg). Subfraction 2A was purified by semi-preparative HPLC (MeCN/H_2_O, 25:75) to afford **4** (4 mg). Fr. 3 was repeatedly chromatographed over MCI-gel CHP-20P (MeOH/H_2_O, 5–45 % with a 5 % increment) and silica gel (CHCl_3_/MeOH/H_2_O, 9:1:0.1–6:4:1) to get subfractions F3A (170 mg) and F3B (180 mg). Subfraction F3A was purified by semi-preparative HPLC (MeCN/H_2_O, 25:75) to give **5** (4 mg). Subfraction F3B was purified by semi-preparative HPLC (MeCN/H_2_O, 14:86) to furnish **1** (14 mg) and **2** (25 mg).

#### Flexuosoid A (**1**)

White amorphous powder; $[\alpha ]_{\text{D}}^{17}$ −41.2 (*c* 0.30, MeOH); UV (MeOH) *λ*_max_ (log *ε*): 203.8 (3.06) nm; IR (KBr) *ν*_max_ 3424, 2965, 2931, 1691, 1629, 1455, 1382, 1263, 1159, 1137, 1058 cm^−1^; ^1^H and ^13^C NMR data, see Tables 1 and 2; ESIMS (pos. ion mode) *m/z* 529 [M + Na]^+^; negative HR ESIMS *m/z* 505.2072 [M − H]^−^ (calcd for C_26_H_33_O_10_, 505.2073).

#### Flexuosoid B (**2**)

White amorphous powder; $[\alpha ]_{\text{D}}^{18}$ −41.7 (*c* 0.68, MeOH); UV (MeOH) *λ*_max_ (log *ε*) 204.0 (3.95) nm; IR (KBr) *ν*_max_ 3424, 2967, 2933, 1695, 1455, 1380, 1257, 1037 cm^−1^; ^1^H and ^13^C NMR data, see Tables 1 and 2; ESIMS (neg. ion mode) *m/z* 583 [M + Cl]^−^; negative HRESIMS *m/z* 547.2182 [M − H]^−^ (calcd for C_28_H_35_O_11_, 547.2179).

#### Phyllanthusmin D (**3**)

White amorphous powder; $[\alpha ]_{\text{D}}^{16}$ +10.5 (*c* 0.07, MeOH); UV (MeOH) *λ*_max_ (log *ε*): 201.0 (4.86), 260.4 (4.94) nm; IR (KBr) *ν*_max_ 3423, 2908, 1742, 1622, 1507, 1480, 1455, 1435, 1386, 1377, 1342, 1264, 1216, 1169, 1080, 1025, 1012 cm^−1^; ^1^H and ^13^C NMR data, see Table 3; ESIMS (neg. ion mode) *m/z* 721 [M + Cl]^−^; negative HRESIMS *m/z* 685.1763 [M − H]^−^ (calcd for C_33_H_33_O_16_, 685.1768).

#### Phyllanthusmin E (**4**)

White amorphous powder; $[\alpha ]_{\text{D}}^{27}$ −48.7 (*c* 0.28, MeOH); UV (MeOH) *λ*_max_ (log *ε*): 203.2 (4.24), 259.0 (4.55) nm; IR (KBr) *ν*_max_ 3442, 2923, 1630, 1535, 1468, 1416, 1385, 1203, 1169, 1128, 1073, 1039 cm^−1^; ^1^H and ^13^C NMR data see Table 3; ESIMS (neg. ion mode) *m/z* 695 [M + Cl]^−^; positive HRESIMS *m/z* 661.1766 [M + H]^+^ (calcd for C_31_H_33_O_16_, 661.1763).

#### Phyllanthusmin F (**5**)

White amorphous powder; $[\alpha ]_{\text{D}}^{22}$ −40.6 (*c* 0.30, MeOH); UV (MeOH) *λ*_max_ (log *ε*): 202.8 (4.41), 262.2 (4.56) nm; IR (KBr) *ν*_max_ 3426, 2925, 1730, 1627, 1507, 1480, 1458, 1435, 1386, 1341, 1265, 1230, 1168, 1075, 1049, 1009 cm^−1^; ^1^H and ^13^C NMR data see Table 4; ESIMS (neg. ion mode) *m/z* 871 [M + Cl]^−^; negative HRESIMS *m/z* 871.2077 [M + Cl]^−^ (calcd for C_38_H_44_O_21_Cl, 871.2063).

#### Acetylation of Compounds **1** and **2**

See Electronic supplementary material.

#### Acid Hydrolysis of Compounds **3–5**

See Electronic supplementary material.

### Antifeedant Activity Assay

The insects, beet armyworm (*Spodoptera exigua*) purchased from the Pilot-Scale Base of Bio-Pesticides, Institute of Zoology, Chinese Academy of Sciences. A dual-choice bioassay modified from previous methods was performed for antifeedant tests [31]. The larvae were reared on an artificial diet in the laboratory under controlled photoperiod (light:dark, 12:8 h) and temperature (25 ± 2 °C). Larvae were star ved 4–5 h prior to each bioassay. Fresh leaf discs were cut from *Brassica chinensis*, using a cork borer (1.1 cm in diameter). Treated leaf discs were painted with 20 μL of acetone solution containing the test compounds, and control leaf discs with the same amount of acetone. After air drying, two tested leaf discs and two control ones were set in alternating position in the same Petri dish (90 mm in diameter), with moistened filter paper at the bottom. Two-thirds of instars were placed at the center of the Petri dish. Five replicates were run for each treatment. After feeding for 24 h, areas of leaf discs consumed were measured. The antifeedant index (AFI) was calculated according to the formula AFI = [(C − T)/(C + T)] × 100, where C and T represent the control and treated leaf areas consumed by the insect. The insect antifeedant potency of the test compound was evaluated in terms of the ED_50_ value (the effective dosage for 50 % feeding reduction) which was determined by Probit analysis for the insect species. The positive control was served as commercial neem oil containing 1 % azadirachtin produced by Kunming Rixin Dachuan Technology Co., Ltd.

### Cytotoxicity Assay

The cytotoxicity of the test compounds on Vero cells was determined by an MTT assay. Vero cells were seeded in 96-well plates at a density of 1 × 10^4^ cells/well, and incubated at 37 °C in a 5 % CO_2_ atmosphere for 24 h, until 90 % or greater confluence of the monolayers was reached. Increasing concentrations of the test compounds were added to cells, with a replicate number of three wells per concentration and 17-AAG served as positive control. After a 2-day incubation period in such conditions, a MTT solution was added (final concentration 0.5 mg/mL) and the plates were incubated for another 4 h to allow formazan production. The solid precipitate was dissolved with DMSO and the absorbance at 570 nm was measured using a 96-well Spectrophotometer (Bio-Rad 550) with a reference wavelength of 630 nm. The cytotoxicity of the test compounds on the ECA109 human esophagus cancer cell and Vero cell lines was determined by an MTT assay. The ECA109 human esophagus cancer cell line was cultured in RPMI 1640 containing 10 % heat inactivated FBS and 100 U/mL penicillin/streptomycin in a humidified incubator in a 5 % CO_2_ atmosphere at 37 °C. Cells (5 × 10^3^/well) were plated in 96-well plates in 100 μL medium, cultured overnight and exposed to a range of concentrations of compounds for 48 h. After the addition of 20 μL MTT solution (5 mg/mL) per well, the plates were incubated for 4 h, the medium were removed, the formazan crystals were solubilized in 100 μL DMSO per well and the absorbance values were read at 570 nm.

### Anti-HSV-1 Assay

Vero cells were seeded in 96-well plates at a density of 1 × 10^4^ cell/well. Confluent cell monolayers were treated with increasing non-cytotoxic concentrations of the plant extract. Four wells were used for each concentration. Afterwards, the cells were infected with HSV-1 (100 TCID_50_), incubated at 37 °C in a 5 % CO_2_ humidified atmosphere and observed daily for cytopathic effect (CPE) using a light microscope. Acyclovir at concentration 20 μg/mL served as positive control. The EC_50_ value was calculated by MTT method.

## Electronic supplementary material

Below is the link to the electronic supplementary material.

The Acetylation of compounds **1** and **2**, and Acid hydrolysis of compounds **3**-**5** sections, the Key ^1^H-^1^H COSY, HMBC and ROESY correlations of **2**, Key HMBC correlations of **4** and **5**, the physicochemical and spectroscopic data of **1a** and **1b**, and the ^1^H and ^13^C NMR, HSQC, ROESY, HMBC and ^1^H-^1^H COSY spectra for compounds **1** - **5**, **1a**, and **1b** are available as supporting information. (PDF 971 kb)
