# Supplementary material for: Highly Oxygenated Limonoids and Lignans from Phyllanthus flexuosus
Source: Nat Prod Bioprospect. 2014 Jul 22;4(4):233–42. doi: 10.1007/s13659-014-0026-2 (PMC4111870; doi:10.1007/s13659-014-0026-2)
Supplement: Supplementary file 1 — The Acetylation of compounds 1 and 2, and Acid hydrolysis of compounds 3-5 sections, the Key 1H-1H COSY, HMBC and ROESY correlations of 2, Key HMBC correlations of 4 and 5, the physicochemical and spectroscopic data of 1a and 1b, and the 1H and 13C NMR, HSQC, ROESY, HMBC and 1H-1H COSY spectra for compounds 1 - 5, 1a, and 1b are available as supporting information. (PDF 971 kb) [file 13659_2014_26_MOESM1_ESM.pdf]

Supporting Information

Highly Oxygenated Limonoids and Lignans from *Phyllanthus flexuosus*

Jian-Qiang Zhao,[a,b,d] Yan-Ming Wang,[a,b,d] Hong-Tao Zhu,[a] Dong Wang,[a] Rong-Rong Cheng,[a] Chong-Ren Yang,[a] Yi-Fei Wang,[c] Min Xu,[a]* and Ying-Jun Zhang[a]*

Affiliation

[a] State Key Laboratory of Phytochemistry and Plant Resources in West China, Kunming Institute of Botany, Chinese Academy of Sciences, Kunming 650201, People's Republic of China

[b] University of Chinese Academy of Sciences, Beijing 100049, People's Republic of China

[c] Guangzhou Jinan Biomedicine Research & Development Center, Guangzhou 510632, People's Republic of China

[d] The authors contributed equally to this paper

Correspondence

Prof. Dr. Ying-Jun Zhang, State Key Laboratory of Phytochemistry and Plant Resources in West China, Kunming Institute of Botany, Chinese Academy of Sciences, Kunming 650201, China. E-mail: zhangyj@mail.kib.ac.cn. Tel/Fax: +86-871-65223235

Assoc. Prof. Dr. Min Xu, State Key Laboratory of Phytochemistry and Plant Resources in West China, Kunming Institute of Botany, Chinese Academy of Sciences, Kunming 650201, China. E-mail: xumin@mail.kib.ac.cn. Tel/ Fax: +86-871-65223235

1. Fig. 1S Key ^{1}H-^{1}H COSY, HMBC and ROESY correlations of flexuosoid B (**2**)
2. Fig. 2S Key HMBC correlations of phyllanthusmins E (**4**) and F (**5**)
3. Acid hydrolysis of compounds **3-5**
4. Acetylation of compounds **1** and **2**
5. Physicochemical and spectroscopic data of compounds **1a** and **1b**
6. Table 1S ^{13}C NMR Spectroscopic Data of compounds **1a** and **1b** (methanol-d_4, δ in ppm)
7. Table 2S ^{1}H NMR Spectroscopic Data of compounds **1a** and **1b** (methanol-d_4, δ in ppm)
8. Fig. S3 ^{1}H NMR spectrum of flexuosoid A (**1**) in methanol-d_4
9. Fig. 4S ^{13}C NMR and DEPT spectra of flexuosoid A (**1**) in methanol-d_4
10. Fig. 5S HSQC spectrum of flexuosoid A (**1**) in methanol-d_4
11. Fig. 6S ^{1}H-^{1}H COSY spectrum for flexuosoid A (**1**) in methanol-d_4
12. Fig. 7S HMBC spectrum of flexuosoid A (**1**) in methanol-d_4
13. Fig. 8S ROESY spectrum of flexuosoid A (**1**) in methanol-d_4
14. Fig. 9S ^{1}H NMR spectrum of flexuosoid B (**2**) in methanol-d_4
15. Fig. 10S ^{13}C NMR and DEPT spectra of flexuosoid B (**2**) in methanol-d_4
16. Fig. 11S HSQC spectrum of flexuosoid B (**2**) in methanol-d_4
17. Fig. 12S ^{1}H-^{1}H COSY spectrum of flexuosoid B (**2**) in methanol-d_4
18. Fig. 13S HMBC spectrum of flexuosoid B (**2**) in methanol-d_4
19. Fig. 14S ROESY spectrum of flexuosoid B (**2**) in methanol-d_4
20. Fig. 15S ^{1}H NMR spectrum of phyllanthusmin D (**3**) in methanol-d_4
21. Fig. 16S ^{13}C NMR and DEPT spectra of phyllanthusmin D (**3**) in methanol-d_4
22. Fig. 17S HSQC spectrum of phyllanthusmin D (**3**) in methanol-d_4
23. Fig. 18S ^{1}H- ^{1}H COSY spectrum of phyllanthusmin D (**3**) in methanol-d_4
24. Fig. 19S HMBC spectrum of phyllanthusmin D (**3**) in methanol-d_4
25. Fig. 20S ROESY spectrum of phyllanthusmin D (**3**) in methanol-d_4
26. Fig. 21S ^{1}H NMR spectrum of phyllanthusmin E (**4**) in methanol-d_4
27. Fig. 22S ^{13}C NMR and DEPT spectra of phyllanthusmin E (**4**) in methanol-d_4
28. Fig. 23S HSQC spectrum of phyllanthusmin E (**4**) in methanol-d_4
29. Fig. 24S HMBC spectrum of phyllanthusmin E (**4**) in methanol-d_4
30. Fig. 25S ROESY spectrum of phyllanthusmin E (**4**) in methanol-d_4
31. Fig. 26S ^{1}H NMR spectrum of phyllanthusmin F (**5**) in pyridine-d_5
32. Fig. 27S ^{13}C NMR and DEPT spectra of phyllanthusmin F (**5**) in pyridine-d_5
33. Fig. 28S HSQC spectrum of phyllanthusmin F (**5**) in pyridine-d_5
34. Fig. 29S ^{1}H-^{1}H COSY spectrum of phyllanthusmin F (**5**) in pyridine-d_5
35. Fig. 30S HMBC spectrum of phyllanthusmin F (**5**) in pyridine-d_5
36. Fig. 31S ROESY spectrum of phyllanthusmin F (**5**) in pyridine-d_5
37. Fig. 32S ^{1}H NMR spectrum of 1,2,3,7,28,30-hexa-acetyl flexuosoid A (**1a**) in methanol-d_4
38. Fig. 33S ^{13}C NMR and DEPT spectra of 1,2,3,7,28,30-hexa-acetyl flexuosoid A (**1a**) in methanol-d_4
39. Fig. 34S HSQC spectrum of 1,2,3,7,28,30-hexa-acetyl flexuosoid A (**1a**) in methanol-d_4
40. Fig. 35S ^{1}H- ^{1}H COSY spectrum of 1,2,3,7,28,30-hexa-acetyl flexuosoid A (**1a**) in methanol-d_4
41. Fig. 36S HMBC spectrum of 1,2,3,7,28,30-hexa-acetyl flexuosoid A (**1a**) in methanol-d_4
42. Fig. 37S ROESY spectrum of 1,2,3,7,28,30-hexa-acetyl flexuosoid A (**1a**) in methanol-d_4
43. Fig. 38S ^{1}H NMR spectrum of 2,3,7,28,30-penta-acetyl flexuosoid A (**1b**) in methanol-d_4
44. Fig. 39S ^{13}C NMR and DEPT spectra of 2,3,7,28,30-penta-acetyl flexuosoid A (**1b**) in methanol-d_4
45. Fig. 40S HSQC spectrum of 2,3,7,28,30-penta-acetyl flexuosoid A (**1b**) in methanol-d_4
46. Fig. 41S ^{1}H- ^{1}H COSY spectrum of 2,3,7,28,30-penta-acetyl flexuosoid A (**1b**) in methanol-d_4
47. Fig. 42S HMBC spectrum of 2,3,7,28,30-penta-acetyl flexuosoid A (**1b**) in methanol-d_4
48. Fig. 43S ROESY spectrum of 2,3,7,28,30-penta-acetyl flexuosoid A (**1b**) in methanol-d_4

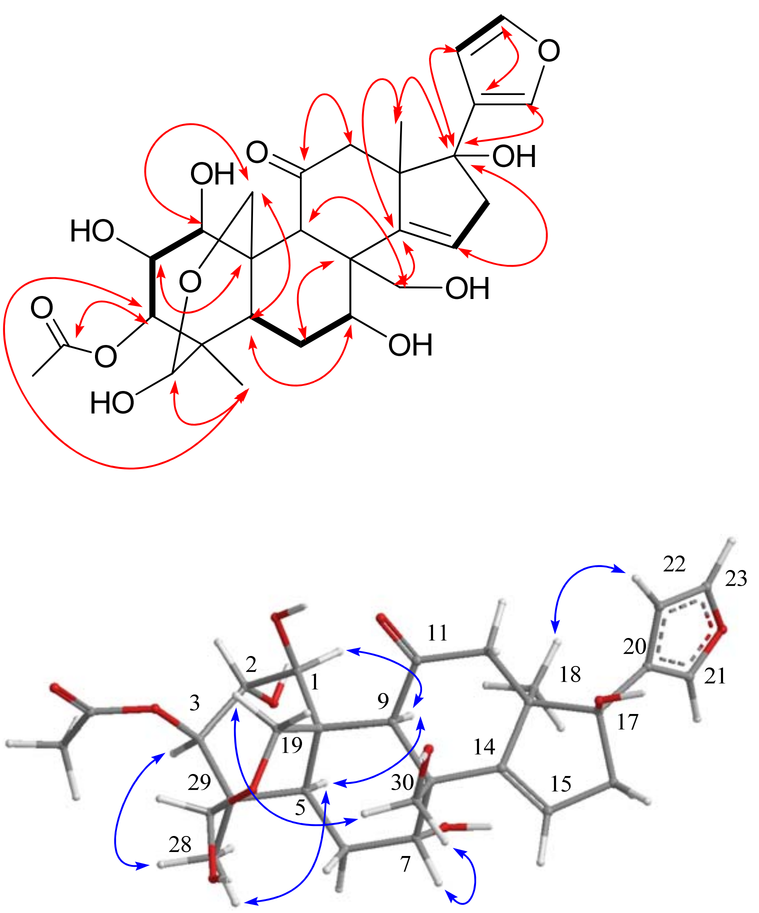

Fig. 1S　Key ^{1}H-^{1}H COSY (━), HMBC (→) and ROESY (↔) correlations of flexuosoid B (2)

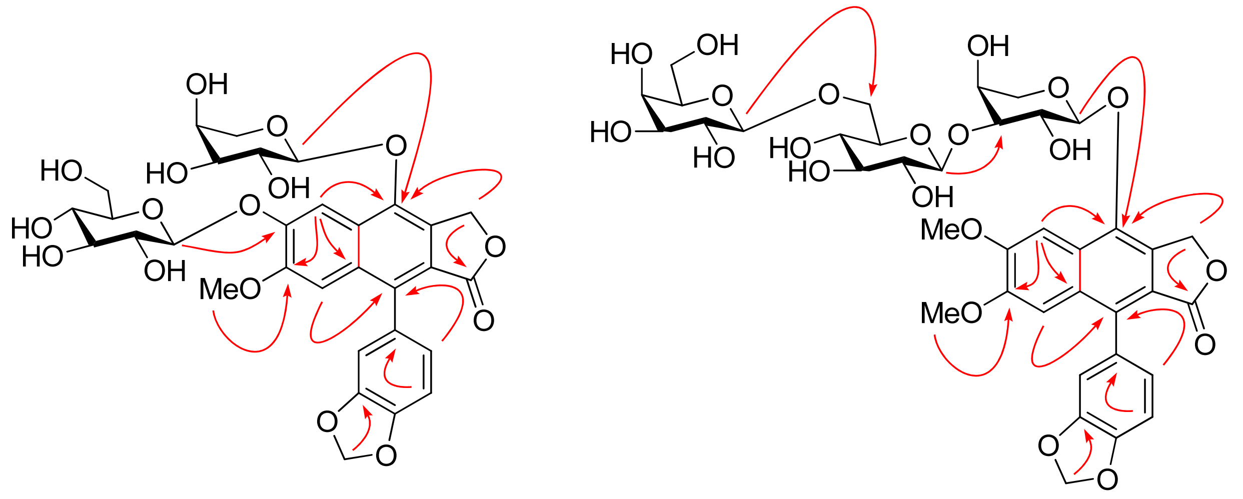

4 **5**

Fig. 2S Key HMBC (→) correlations of phyllanthusmins E (4) and F (5)

Acid hydrolysis of compounds 3-5

Compounds **3-5** (each 3 mg) in 2 M HCl-dioxane (1:1, v/v, 5 mL) were heated at 85°C in a water bath for 8 h, respectively. The reaction mixtures were partitioned between H_2O and $CHCl_3$ (2 mL × 3) four times. The aqueous layer was neutralized with 2 M NaOH and then dried to give a saccharide mixture. Solutions of the sugar residues of these compounds in pyridine (2 mL) were added to L-cysteine methyl ester hydrochloride (1.5 mg) and kept at 60°C for 1 h. Trimethylsilylimidazole (1.5 mL) was added to the reaction mixtures, and they kept at 60°C for 30 min. The supernatants (4 μL) were analyzed by GC, respectively. The saccharides of compounds **3-5** were determined by comparison of the retention times of the corresponding derivatives with those of the standard saccharides, of which retention times of D-galactose, L-arabinose and D-glucose are 22.229, 16.591, and 21.746 min, respectively. The monosaccharide of **3** was determinedto be L-arabinose (16.588 min), while the monosaccharides of **4** were L-arabinose (16.459 min) and D-glucose (21.787 min). The monosaccharides of **5** were determined to be L-arabinose (16.233 min), D-glucose (21.516 min) and D-galactose (22.141 min).

Acetylation of compounds 1 and 2

Compound **1** (9.2 mg, 0.0182 mmol) was added to the mixture of DMAP (2.2 mg, 0.0182 mmol) and Et_3N (76 μl, 0.545mmol) in 2 ml anhydrous DCM in ice-bath, and then Ac_2O (51.5 μl, 0.0545 mmol) was added to the mixture under Ar. The reaction mixture was stirred at 0°C. After 24 h the solution was evaporated to dryness. The residue was applied to semi-preparative HPLC to furnish compounds **1a** (3.9 mg) in 30% yield and **1b** (3.6 mg) in 27% yield. In the same way, compound **2** (18.0 mg, 0.0328 mmol) was acetylated, to give the same adducts **1a** (7.6 mg) in 31% yield and **1b** (7.0 mg) in 28% yield, as those of **1**.

1,2,3,7,29,30-hexa-acetyl flexuosoid A (1a): white amorphous powder; $[\alpha]_D^{16.4}$ -53.4 (*c* 0.09, MeOH); UV (MeOH) λ_{max} (log ε) 203.6 (3.93), 260.4 (2.76) nm; IR (KBr) ν_{max} 3468, 2971, 2930, 1747, 1374, 1230, 1256, 1054 cm^{-1}; ^1H and ^{13}C NMR data, see Tables **1S** and **2S**; EI MS (pos. ion mode) *m/z* 758 $[M]^+$; positive HR EIMS *m/z* 758.2791 $[M]^+$ (calcd for $C_{38}H_{46}O_{16}$, 758.2786).

2,3,7,29,30-penta-acetyl flexuosoid A (1b): white amorphous powder; $[\alpha]_D^{16.6}$ -62.0 (*c* 0.11, MeOH); UV (MeOH) λ_{max} (log ε) 203.8 (3.93) nm; IR (KBr) ν_{max} 3478, 2971, 2934, 1745, 1432, 1231, 1054 cm^{-1}; ^1H and ^{13}C NMR data, see Tables **1S** and **2S**; EI MS (neg. ion mode) *m/z* 716 $[M]^+$; positive HR EIMS *m/z* 716.2667 $[M]^+$ (calcd for $C_{36}H_{44}O_{15}$, 716.2680).

Table **1S** ^{13}C NMR Spectroscopic Data of **1a** and **1b** (methanol-d_4, δ in ppm)

	**1a**[a]	**1b**[a]
1	73.7, CH	71.3, CH
2	68.7, CH	70.7, CH
3	73.2, CH	73.6, CH
4	41.7, C	41.7, C
5	31.2, CH	30.4, CH
6	24.0, CH_2	24.1, CH_2
7	71.8, CH	71.9, CH
8	48.0, C	47.7, C
9	46.6, CH	46.7, CH
10	42.0, C	43.1, C
11	211.3, C	212.7, C
12	44.1, CH_2	44.0, CH_2
13	52.7, C	53.0, C
14	151.1, C	151.8, C
15	122.6, CH	122.0, CH
16	44.0, CH_2	44.2, CH_2
17	84.2, C	83.1, C
18	26.9, CH_3	26.5, CH_3
19	64.4, CH_2	65.2, CH_2
20	129.3, C	129.4, C
21	141.2, CH	141.1, CH
22	110.6, CH	110.7, CH
23	144.5, CH	144.4, CH
28	18.6, CH_3	18.7, CH_3
29	94.8, CH	95.1, CH
30	72.6, CH_2	72.8, CH_2
<u>CO</u>Me	171.7, C	171.2, C
	171.2, C	171.5, C
	171.5, C	172.9, C
	170.8, C	171.8, C
	171.0, C	171.8, C
	172.1, C	
CO<u>Me</u>	20.5, CH_3	20.5, CH_3
	20.6, CH_3	20.6, CH_3
	20.8, CH_3	20.8, CH_3
	20.8, CH_3	20.8, CH_3
	21.0, CH_3	21.0, CH_3
	21.1, CH_3	

[a]Data were measured at 150 MHz.

Table **2S** ^{1}H NMR Spectroscopic Data of **1a** and **1b** (methanol-d_4, δ in ppm)

	**1a**[a]	**1b**[a]
1	5.86, d (4.4)	4.52[b]
2	5.92, t (4.4)	5.79, t (4.7)
3	5.41, d (4.4)	5.45, d (4.7)
5	2.81, dd (14.0, 4.0,)	2.78, dd (14.2, 4.0)
6	2.13[b]	2.15[b]
	1.88, dt (14.7, 4.0)	1.90, dt (14.8, 3.8)
7	5.44, brs	5.44, brs
9	3.56, s	4.28, s
12	3.45, d (19.4)	3.46, d (19.0)
	1.95[b]	2.08[b]
15	5.67, brs	5.67, d (1.9)
16	3.15, d (16.5)	3.19, dd (16.5, 1.4)
	2.46, dd (16.5, 3.4)	2.49, dd (16.5, 3.5)
18	0.97, s	1.09, s
19	4.65[b]	4.55[b]
	4.11, d (13.2)	4.11, d (12.9)
21	7.53, s	7.56, s
22	6.41, s	6.46, d (1.8)
23	7.46, s	7.50, t (1.8)
28	0.83, s	0.82, s
29	5.81, s	5.82, s
30	4.44, d (11.4)	4.49, d (11.3)
	4.24, d (11.4)	4.24, d (11.3)
COMe	1.90, s	2.01, s
	1.96, s	2.05, s
	2.08, s (x 2)	2.10, s
	2.11, s	2.14, s
	2.14, s	2.16, s

[a] Data were measured at 500 MHz. [b] Overlapping ^{1}H NMR signals are reported without designated multiplicity.

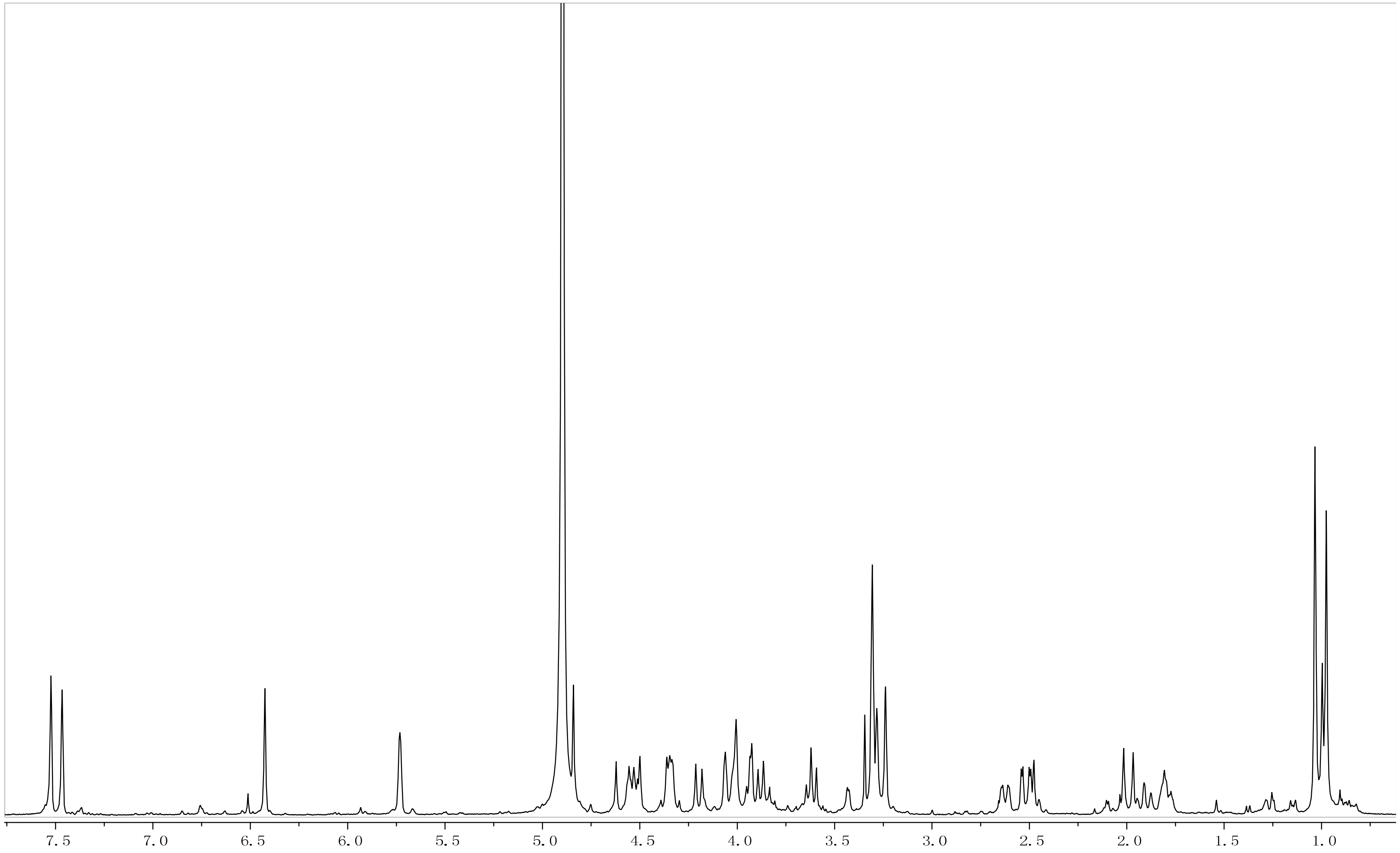

Fig. 3S **^{1}H NMR spectrum of flexuosoid A (1) in methanol-*d*[4]**

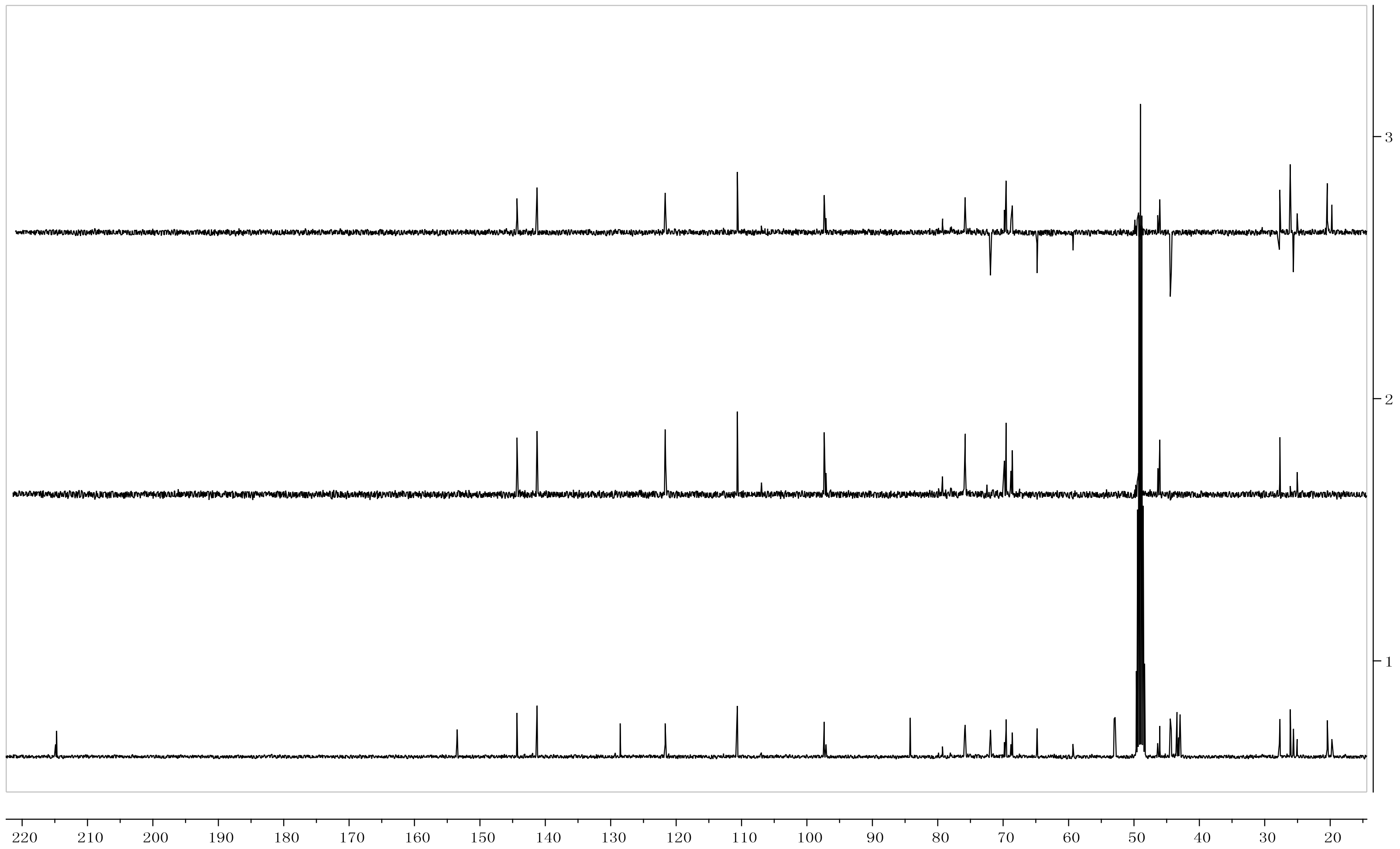

Fig. 4S ^{13}C NMR and DEPT spectra of flexuosoid A (1) in methanol-d_4

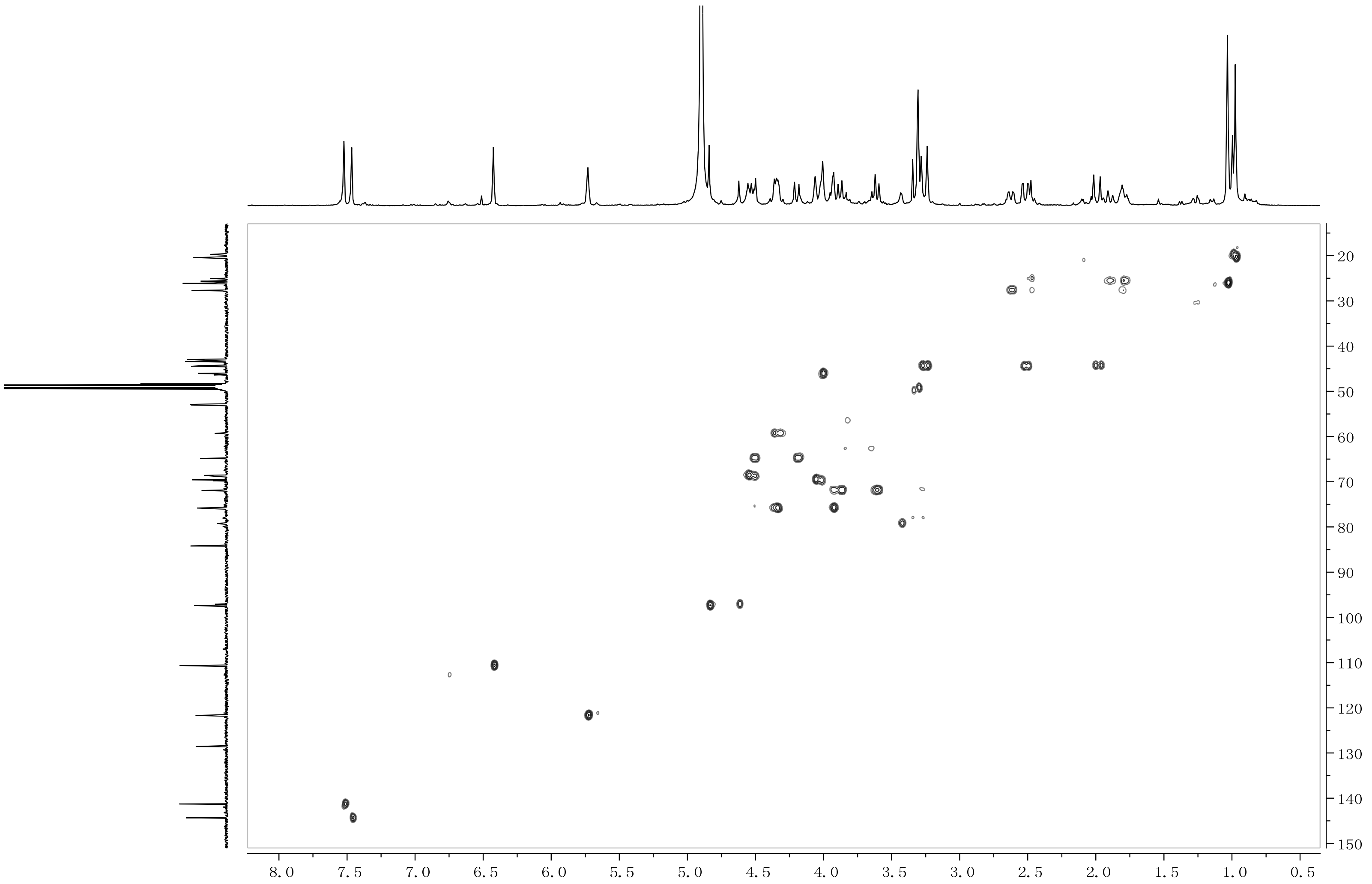

Fig. 5S HSQC spectrum of flexuosoid A (1) in methanol-*d*$_4$

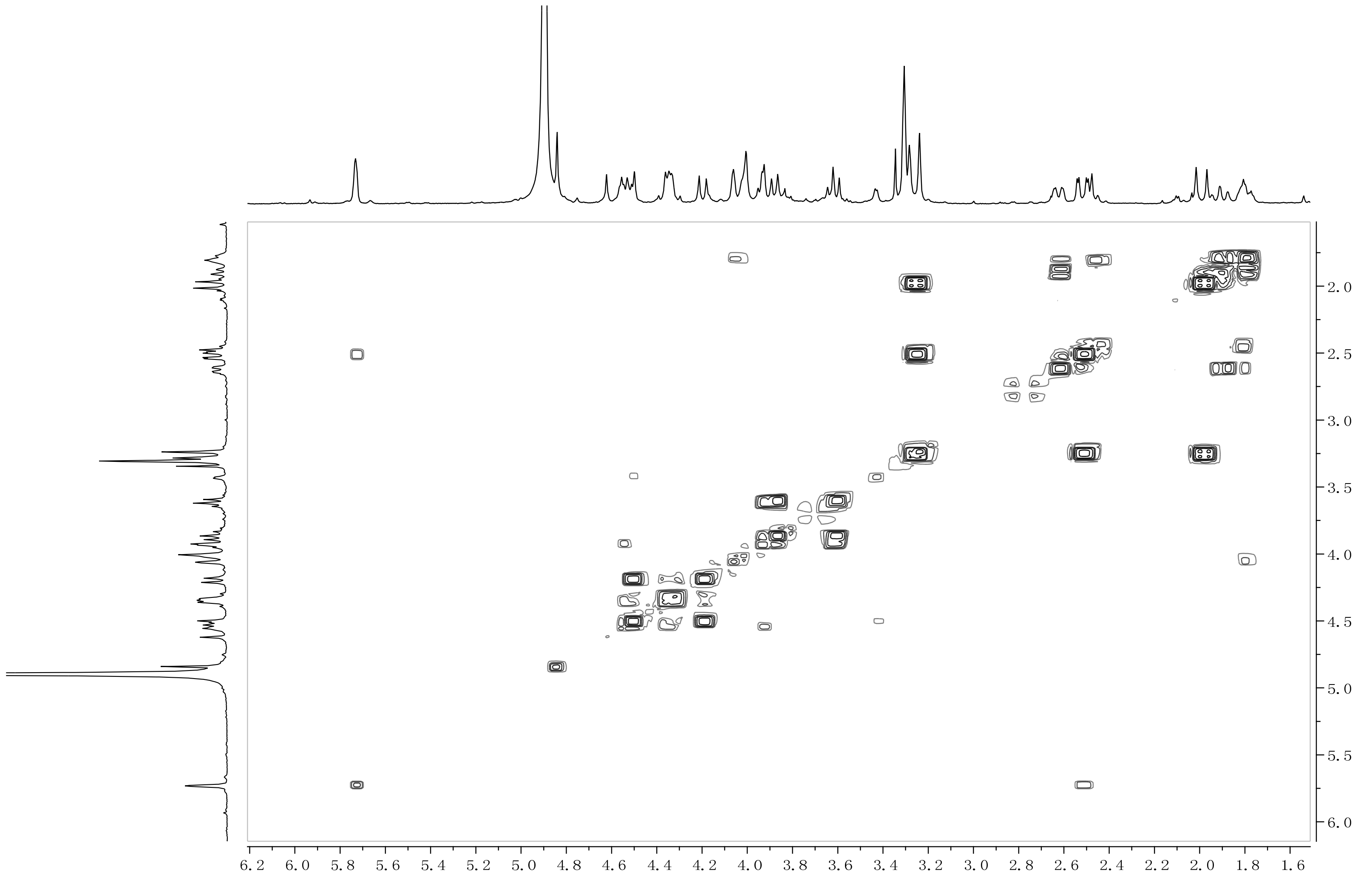

Fig. 6S ^{1}H-^{1}H COSY spectrum for flexuosoid A (1) in methanol-d_4

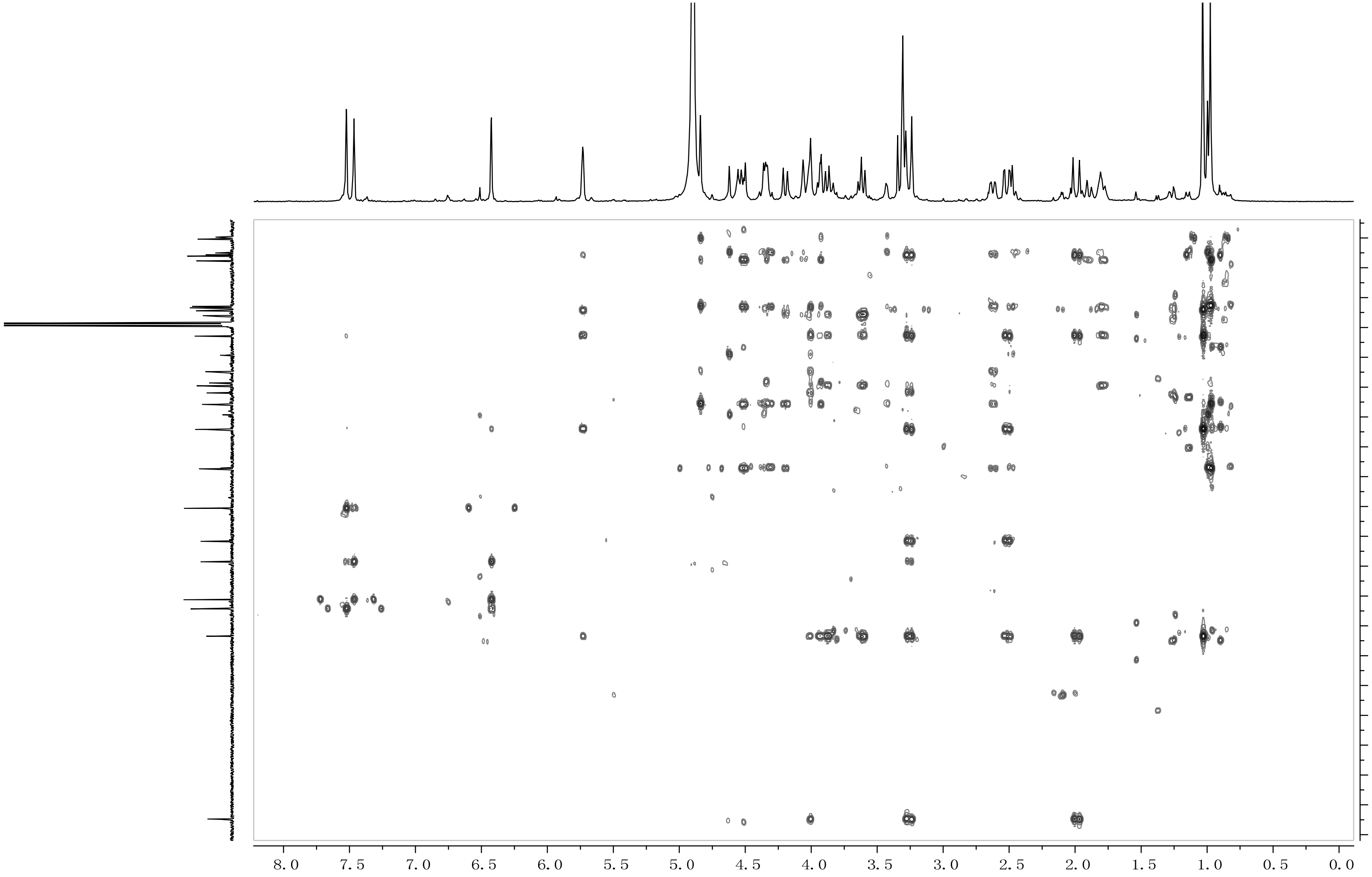

Fig. 7S HMBC spectrum of flexuosoid A (1) in methanol-d_4

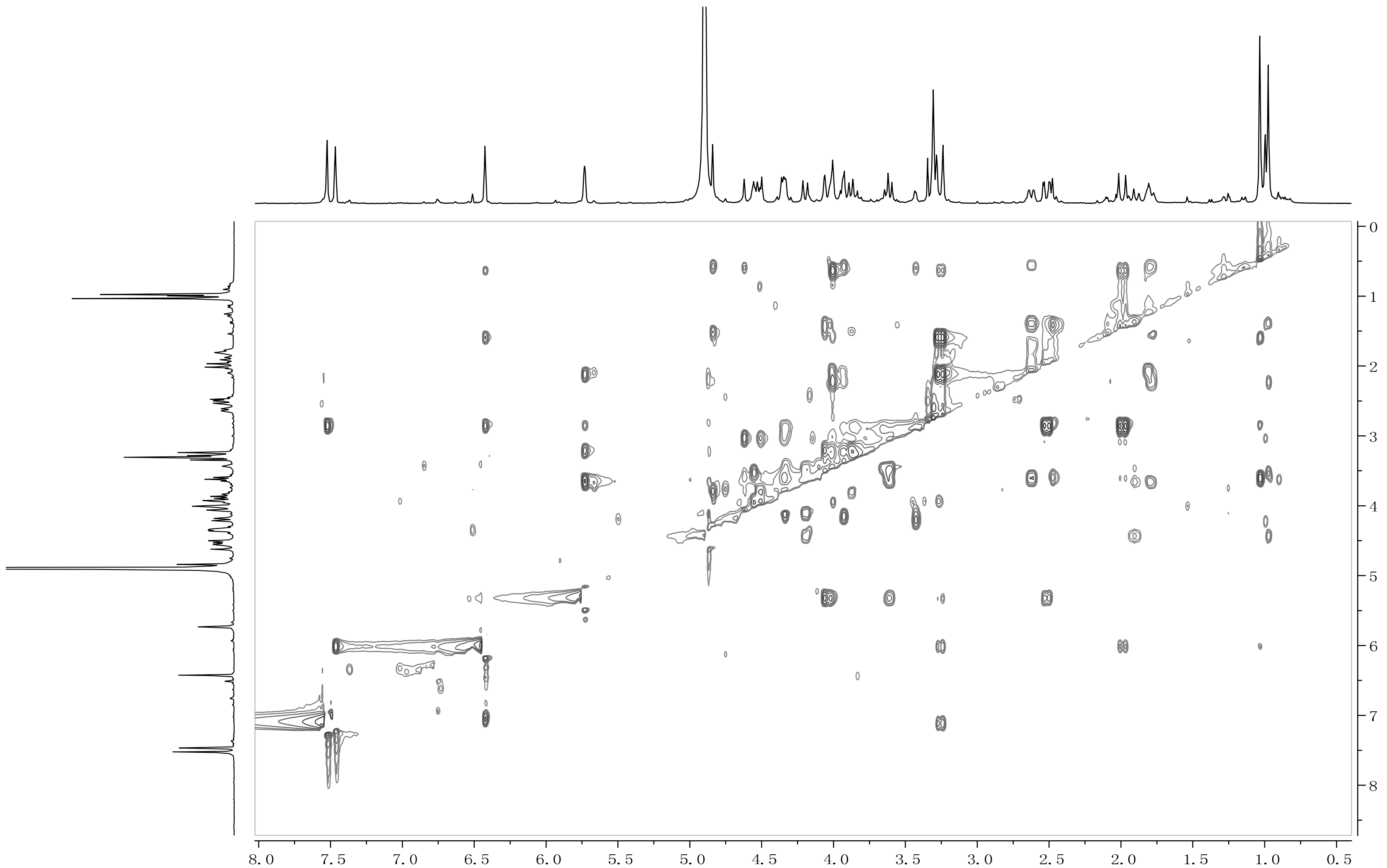

Fig. 8S ROESY spectrum of flexuosoid A (1) in methanol-*d*$_4$

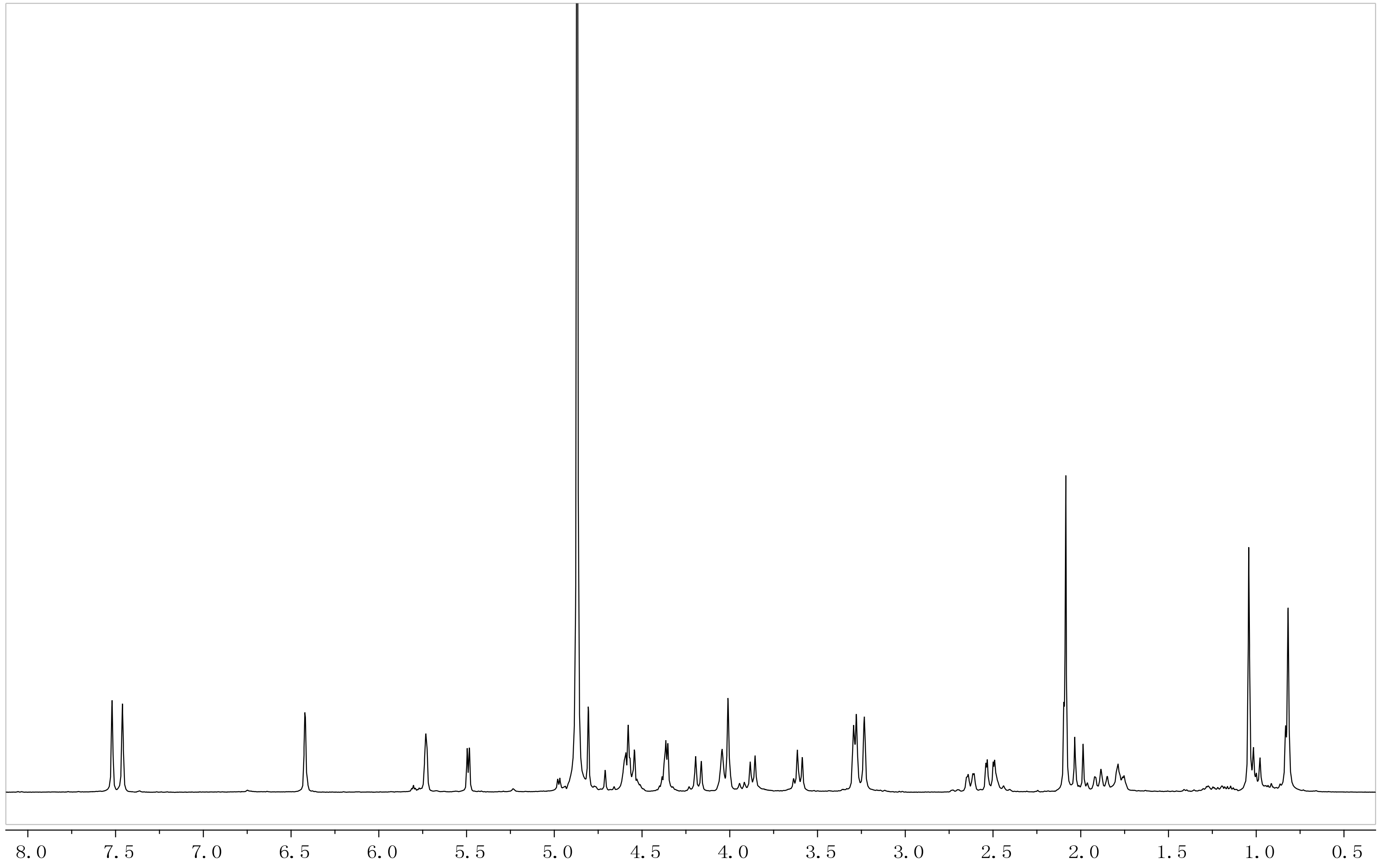

Fig. 9S ^{1}H NMR spectrum of flexuosoid B (2) in methanol-d_4

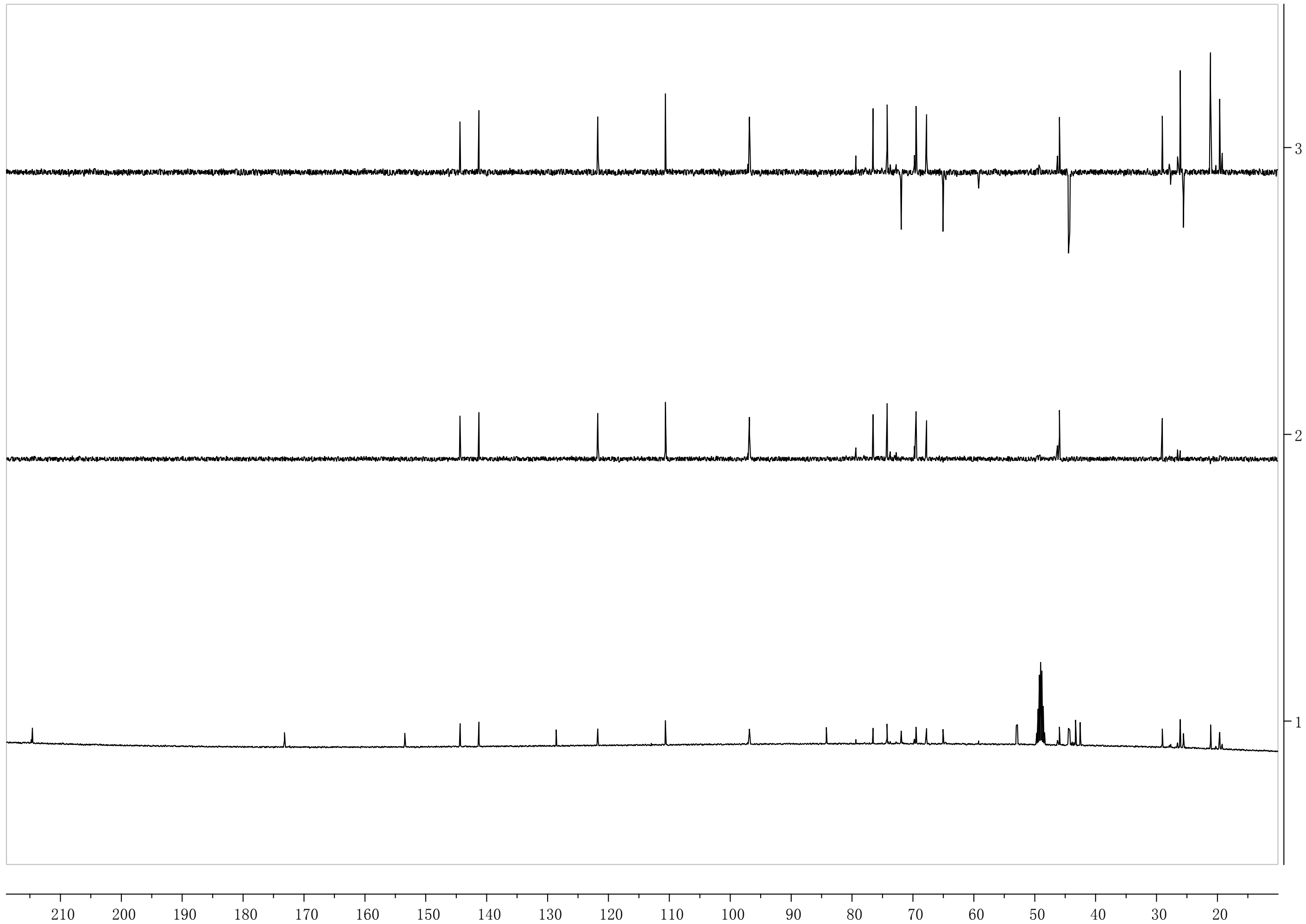

Fig. 10S ^{13}C NMR and DEPT spectra of flexuosoid B (2) in methanol-d_4

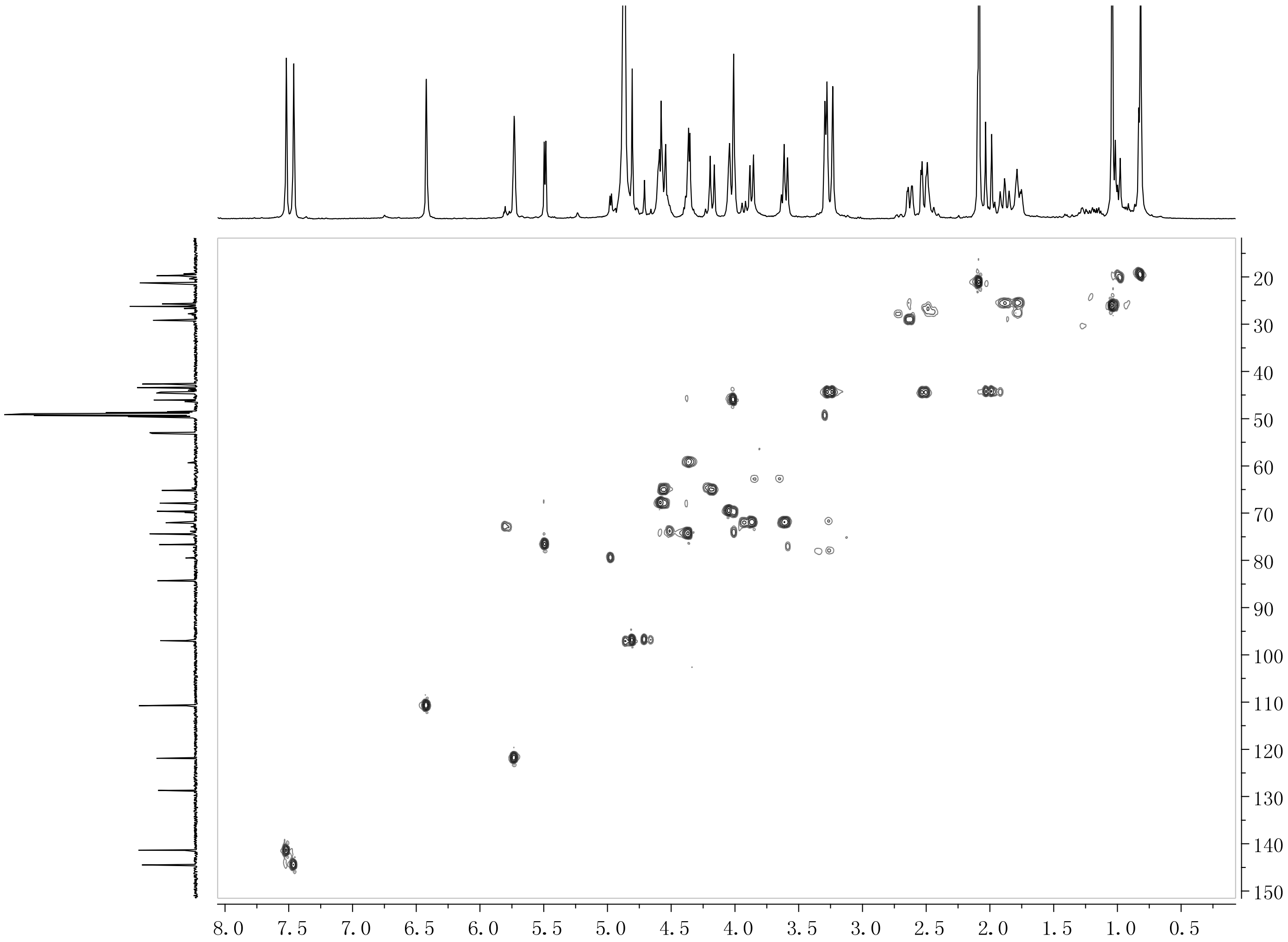

Fig. 11S HSQC spectrum of flexuosoid B (2) in methanol-d_4

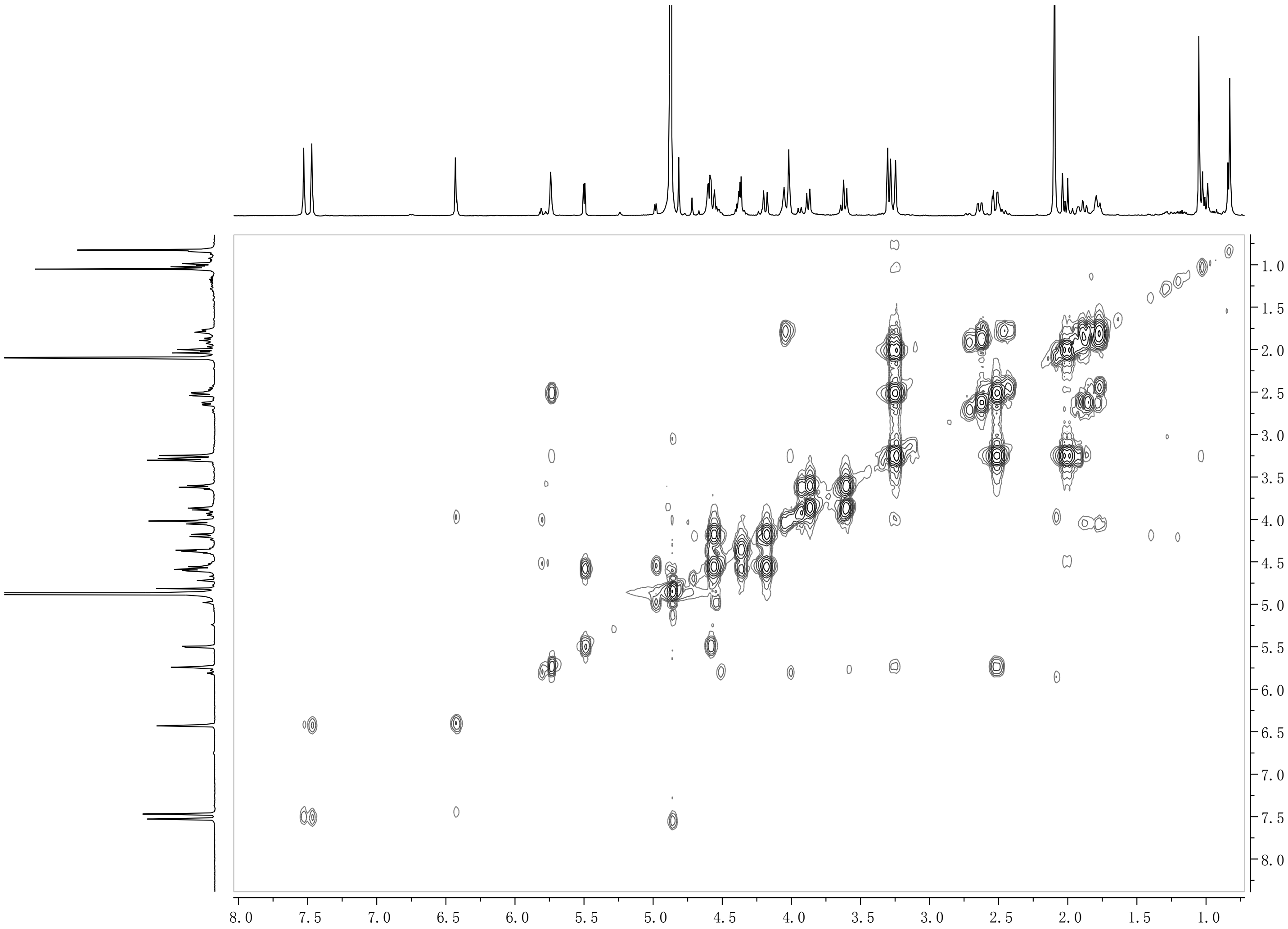

Fig. 12S ^{1}H-^{1}H COSY spectrum of flexuosoid B (2) in methanol-d_4

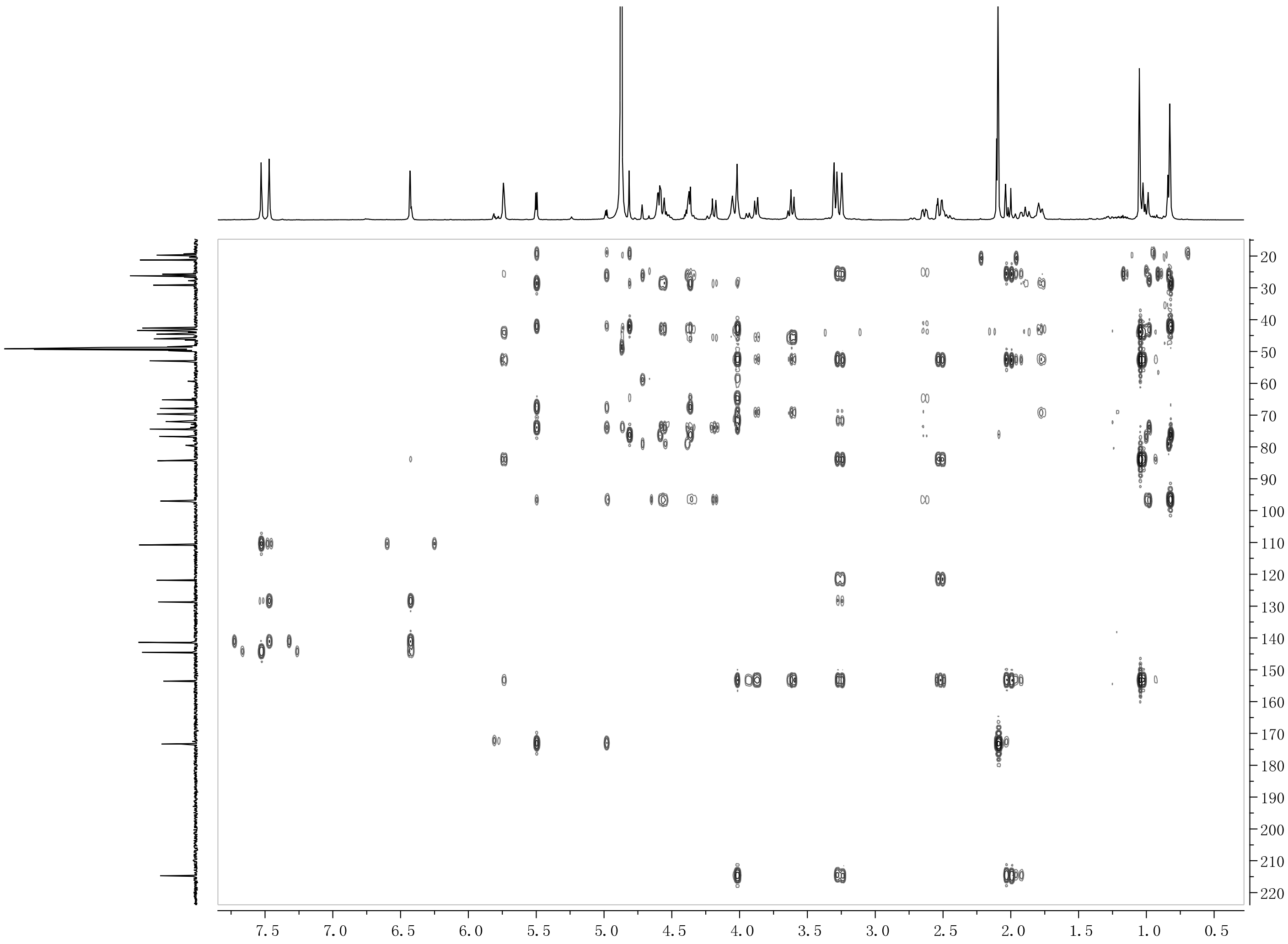

Fig. 13S HMBC spectrum of flexuosoid B (2) in methanol-d_4

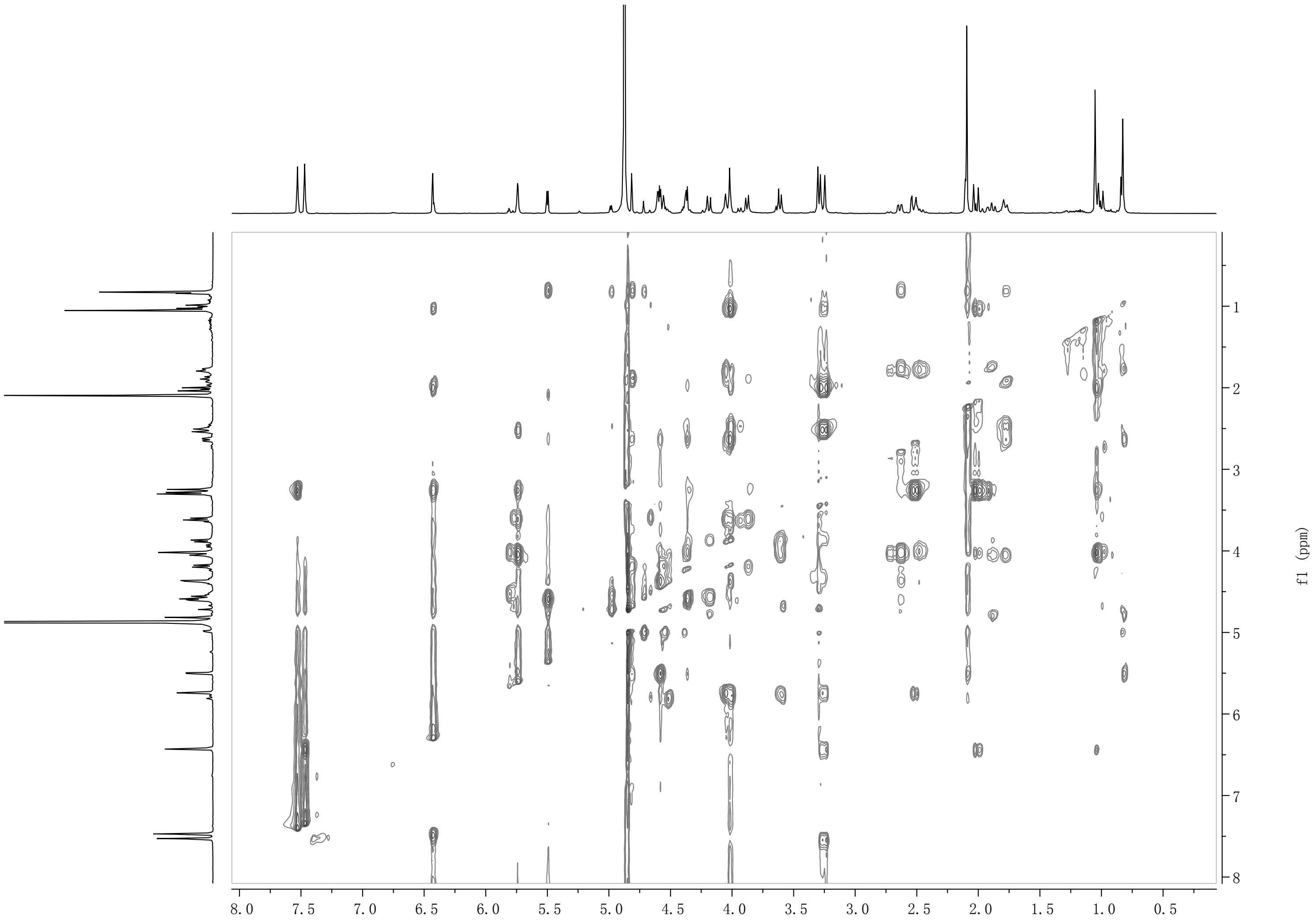

Fig. 14S ROESY spectrum of flexuosoid B (2) in methanol-d_4

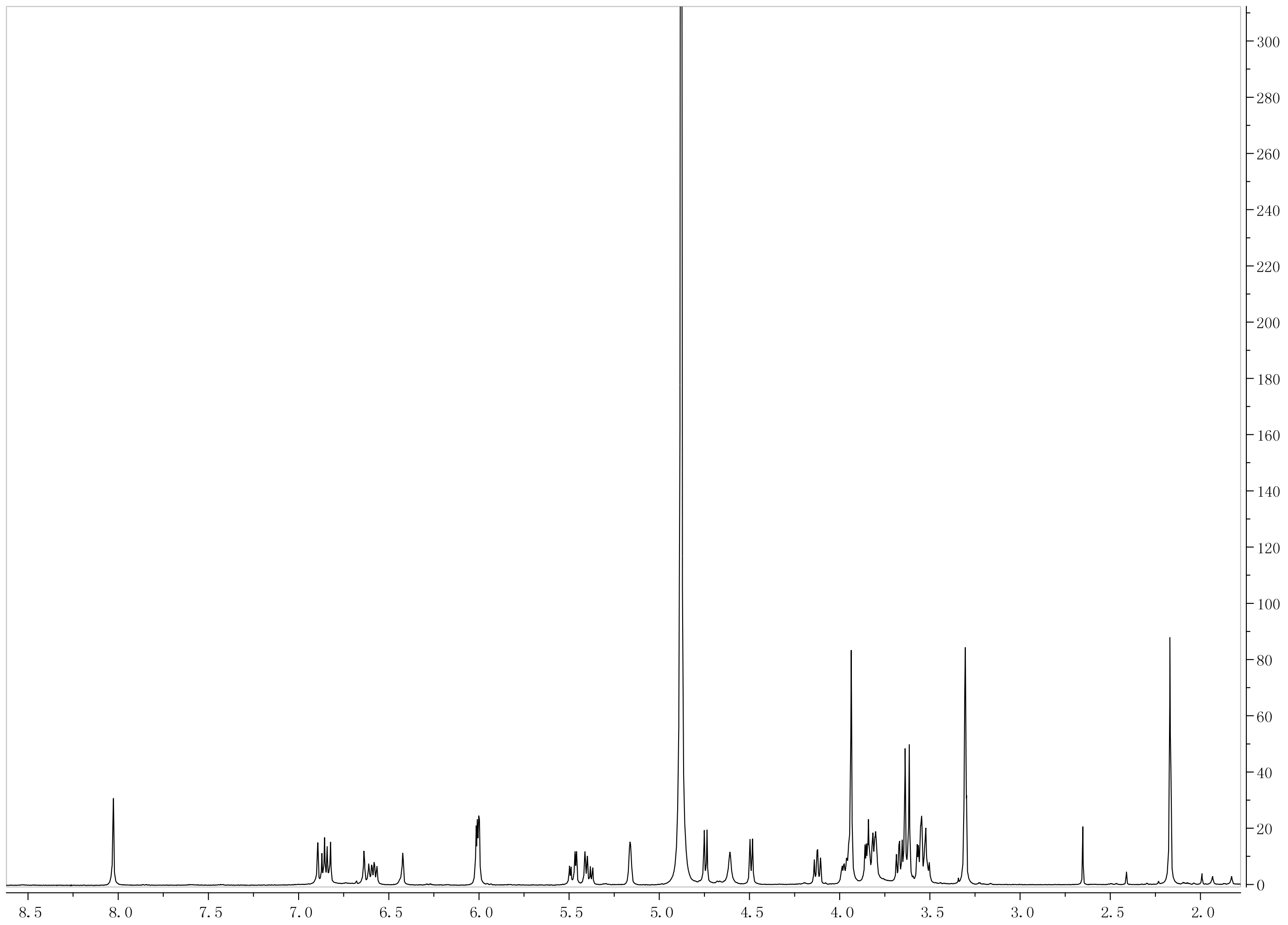

Fig. 15S ^{1}H NMR spectrum of phyllanthusmin D (3) in methanol-d_4

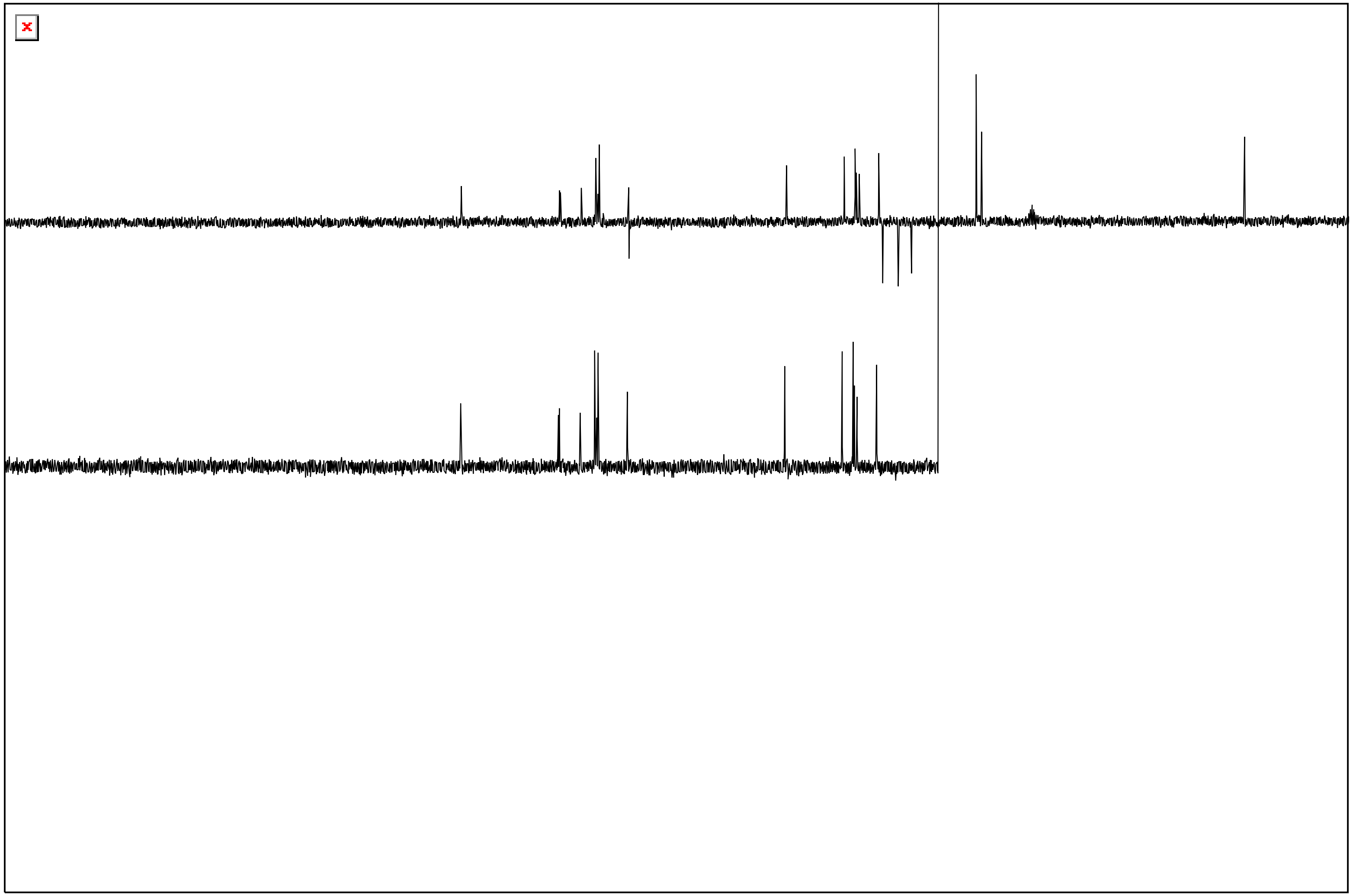

Fig. 16S ^{13}C NMR and DEPT spectra of phyllanthusmin D (3) in methanol-d_4

Fig. 17S HSQC spectrum of phyllanthusmin D (3) in methanol-d_4

Fig. 18S ^{1}H- ^{1}H COSY spectrum of phyllanthusmin D (3) in methanol-d_4

Fig. 19S HMBC spectrum of phyllanthusmin D (3) in methanol-d_4

Fig. 20S ROESY spectrum of phyllanthusmin D (3) in methanol-d_4

Fig. 21S ^{1}H NMR spectrum of phyllanthusmin E (4) in methanol-*d_4*

Fig. 22S ^{13}C NMR and DEPT spectra of phyllanthusmin E (4) in methanol-*d_4*

Fig. 23S HSQC spectrum of phyllanthusmin E (4) in methanol-*d_4*

Fig. 24S HMBC spectrum of phyllanthusmin E (4) in methanol-*d_4*

Fig. 25S ROESY spectrum of phyllanthusmin E (4) in methanol-d_4

Fig. 26S ^{1}H NMR spectrum of phyllanthusmin F (5) in pyridine-*d_5*

Fig. 27S ^{13}C NMR and DEPT spectra of phyllanthusmin F (5) in pyridine-*d_5*

Fig. 28S HSQC spectrum of phyllanthusmin F (5) in pyridine-*d_5*

Fig. 29S ^{1}H-^{1}H COSY spectrum of phyllanthusmin F (5) in pyridine-*d_5*

Fig. 30S HMBC spectrum of phyllanthusmin F (5) in pyridine-d_5

Fig. 31S ROESY spectrum of phyllanthusmin F (5) in pyridine-d_5

Fig. 32S ^{1}H NMR spectrum of 1,2,3,7,28,30-hexa-acetyl flexuosoid A (1a) in methanol-d_4

Fig. 33S ^{13}C NMR and DEPT spectra of 1,2,3,7,28,30-hexa-acetyl flexuosoid A (1a) in methanol-d_4

Fig. 34S HSQC spectrum of 1,2,3,7,28,30-hexa-acetyl flexuosoid A (1a) in methanol-d_4

Fig. 35S ^{1}H- ^{1}H COSY spectrum of 1,2,3,7,28,30-hexa-acetyl flexuosoid A (1a) in methanol-d_4

Fig. 36S HMBC spectrum of 1,2,3,7,28,30-hexa-acetyl flexuosoid A (1a) in methanol-d_4

Fig. 37S ROESY spectrum of 1,2,3,7,28,30-hexa-acetyl flexuosoid A (1a) in methanol-d_4

Fig. 38S ^{1}H NMR spectrum of 2,3,7,28,30-penta-acetyl flexuosoid A (1b) in methanol-d_4

Fig. 39S ^{13}C NMR and DEPT spectra of 2,3,7,28,30-penta-acetyl flexuosoid A (1b) in methanol-d_4

Fig. 40S HSQC spectrum of 2,3,7,28,30-penta-acetyl flexuosoid A (1b) in methanol-d_4

Fig. 41S ^{1}H- ^{1}H COSY spectrum of 2,3,7,28,30-penta-acetyl flexuosoid A (1b) in methanol-d_4

Fig. 42S HMBC spectrum of 2,3,7,28,30-penta-acetyl flexuosoid A (1b) in methanol-*d_4*

Fig. 43S ROESY spectrum of 2,3,7,28,30-penta-acetyl flexuosoid A (1b)) in methanol-*d_4*
